# Adverse effects of Z-drugs for sleep disturbance in people living with dementia: a population-based cohort study

**DOI:** 10.1186/s12916-020-01821-5

**Published:** 2020-11-24

**Authors:** Kathryn Richardson, Yoon K. Loke, Chris Fox, Ian Maidment, Robert Howard, Nicholas Steel, Antony Arthur, Penelope J. Boyd, Clare Aldus, Clive Ballard, George M. Savva

**Affiliations:** 1 Norwich Medical School, University of East Anglia, Norwich, NR4 7TJ UK; 2 School of Life and Health Sciences, Aston University, Birmingham, B4 7ET UK; 3 Division of Psychiatry, UCL Division of Psychiatry, University College London, Maple House, 149 Tottenham Court Road, London, W1T 7NF UK; 4 School of Health Sciences, University of East Anglia, Norwich, NR4 7TJ UK; 5 Medical School, University of Exeter, Exeter, EX1 2LU UK; 6 Quadram Institute Bioscience, Norwich Research Park, Norwich, NR4 7UQ UK

**Keywords:** Dementia, Alzheimer disease, Cohort studies, Sleep initiation and maintenance disorders, Benzodiazepines, Zolpidem, Hip fractures, Accidental falls

## Abstract

**Background:**

Sleep disturbance is common in dementia and often treated with Z-drugs (zopiclone, zaleplon, and zolpidem). While some observational studies suggest that Z-drugs are associated with adverse events such as falls and fracture risks in older people, this has not been studied in dementia.

**Methods:**

We used data from 27,090 patients diagnosed with dementia between January 2000 and March 2016 from the Clinical Practice Research Datalink linked to Hospital Episodes Statistics data in England. We compared adverse events for 3532 patients newly prescribed Z-drugs by time-varying dosage to (1) 1833 non-sedative-users with sleep disturbance; (2) 10,214 non-sedative-users with proximal GP consultation matched on age, sex, and antipsychotic use; and (3) 5172 patients newly prescribed benzodiazepines. We defined higher dose Z-drugs and benzodiazepines as prescriptions equivalent to ≥ 7.5 mg zopiclone or > 5 mg diazepam daily. Cox regression was used to estimate hazard ratios (HRs) for incident fracture, hip fracture, fall, mortality, acute bacterial infection, ischaemic stroke/transient ischaemic attack, and venous thromboembolism over a 2-year follow-up, adjusted for demographic- and health-related covariates.

**Results:**

The mean (SD) age of patients was 83 (7.7) years, and 16,802 (62%) were women. Of 3532 patients prescribed Z-drugs, 584 (17%) were initiated at higher doses. For patients prescribed higher dose Z-drugs relative to non-users with sleep disturbance, the HRs (95% confidence interval) for fractures, hip fractures, falls, and ischaemic stroke were 1.67 (1.13–2.46), 1.96 (1.16–3.31), 1.33 (1.06–1.66), and 1.88 (1.14–3.10), respectively. We observed similar associations when compared to non-sedative-users with proximal GP consultation. Minimal or inconsistent excess risks were observed at ≤ 3.75 mg zopiclone or equivalent daily, and for mortality, infection, and venous thromboembolism. We observed no differences in adverse events for Z-drugs compared to benzodiazepines, except lower mortality rates with Z-drugs (HR [95% confidence interval] of 0.73 [0.64–0.83]).

**Conclusions:**

Higher dose Z-drug use in dementia is associated with increased fracture and stroke risks, similar or greater to that for higher dose benzodiazepines. Higher dose Z-drugs should be avoided, if possible, in people living with dementia, and non-pharmacological alternatives preferentially considered. Prescriptions for higher dose Z-drugs in dementia should be regularly reviewed.

**Trial registration:**

ENCePP e-register of studies, EUPAS18006

## Background

Around 60% of people living with dementia (PlwD) are affected by sleep disturbance [1, 2], including insomnia, fragmented night-time sleep, night-time wandering, or excessive day sleep [3]. Sleep disturbance affects the quality of life of PlwD and their informal carers and often leads to care home admission [4].

Benzodiazepines are frequently used for insomnia in PlwD and act by binding to gamma-aminobutyric acid, an inhibitory neurotransmitter [5]. Benzodiazepines are associated with a range of adverse side-effects including cognitive impairment, daytime sedation, tolerance, dependence, and falls [6–9]. Z-drugs (zaleplon, zopiclone, eszopiclone, and zolpidem), a class of non-benzodiazepine gamma-aminobutyric acid agonists, have shorter half-lives and were originally believed to be safer than benzodiazepines, but their adverse effects are increasingly recognised [10, 11]. Observational studies report Z-drugs are associated with increased risks of falls, fractures, stroke, mortality, and infection in older adults [12–16]. However, these studies are generally subject to residual confounding by sleep disturbance and comorbidity. Studies have also not typically examined how the timing and dosages of Z-drugs might modify risk. More importantly, the adverse effects of Z-drugs have rarely been studied in PlwD, where these side-effects can be particularly catastrophic [17]. A recent Cochrane review found insufficient evidence to guide drug treatment of sleep problems in dementia [18], despite wide-scale prescribing [19]. In addition, the effectiveness of Z-drugs to improve sleep in older people is uncertain and considered limited [11], with cognitive behaviour therapy demonstrated to be more effective at managing insomnia than zopiclone in older adults [20].

We examined the association between first Z-drug prescription and subsequent risk of falls, fractures, mortality, infection, ischaemic stroke, and venous thromboembolism in PlwD. To reduce confounding, we compared Z-drug users with (1) non-users with sleep disturbance, (2) non-users with a proximal GP consultation, and (3) new benzodiazepine users.

## Methods

### Study design

We performed a series of cohort studies, using data from the Clinical Practice Research Datalink (CPRD) linked to Hospital Episode Statistics (HES), Office for National Statistics (ONS) mortality data, and Index of Multiple Deprivation data in England. CPRD collates all diagnoses, referrals, and prescribing records for over 11.3 million patients broadly representative of the UK population [21]. Diagnosis information is electronically entered as UK Read codes [22]. HES records all diagnoses made during a hospital admission (coded using the International classification of diseases 10th revision [ICD-10]) and demographic information [23], and ONS provides date and cause of death (ICD-10 coded) [24]. The Index of Multiple Deprivation combines a number of indicators of housing, employment, income, education, and environment at the general practice level [25]. CPRD obtained ethical approval from a National Research Ethics Service Committee, allowing researchers to access anonymised data for observational studies upon approval from an Independent Scientific Advisory Committee.

### Study population

We defined dementia patients by record of a dementia diagnosis in CPRD (codes in Additional file 1) or HES (ICD-10 F00-F03, G30, G31.0 or G31.1) *or* prescription of a cognitive enhancer (i.e. memantine, donepezil, rivastigmine, or galantamine), occurring after January 2000 when aged ≥ 55 years. We excluded patients with < 3 months of ‘up-to-standard’ (research quality) data or with severe mental illness or Down syndrome before dementia diagnosis (codes in Additional file 1) [21].

### Exposures

Our primary exposure was new prescription of Z-drugs. We considered three comparator groups to reduce confounding [26]. The primary comparator was record of sleep disturbance without sedative-hypnotic prescription. Secondary comparators were (a) non-users at a proximal GP consultation and (b) new benzodiazepine users. To facilitate these comparisons, three main cohorts were constructed, with their index date as the first date after dementia diagnosis of (a) prescription for a Z-drug (World Health Organization’s Anatomic Therapeutic Classification [ATC] system category N05CF), (b) prescription of any benzodiazepine (ATC N05BA or N05CD except midazolam injection), and (c) code for sleep disturbance (codes in Additional file 1) but without a concurrent sedative-hypnotic (ATC N05C or N05BA) prescription.

Two additional cohorts were created by matching the Z-drug and BZD cohorts on age, sex, and antipsychotic use, to non-users (not prescribed sedative-hypnotics) with proximal GP consultation. We matched three non-users to each Z-drug or benzodiazepine user, without replacement, and assigned an index date as the closest GP consultation within 1 month of the corresponding Z-drug or benzodiazepine index date. Patients could be members of different cohorts over time.

Exclusion criteria for all cohorts were (1) < 12 months data history; (2) sedative-hypnotic prescription in the last 12 months; (3) prior diagnosis of sleep apnoea, sleep-related respiratory failure, or alcohol abuse (codes in Additional file 1); (4) prescription of multiple sedative-hypnotics; and (5) newly prescribed antipsychotics or low-dose tricyclic or related antidepressants (≤ 25 mg amitriptyline or ≤ 50 mg trazodone per day). We additionally performed a separate validation study on the accuracy of our patient selection (details in Additional file 2). In summary, GP practices confirmed dementia diagnoses in 96% of cases; however, uncertainty was raised regarding the accuracy of those identified with sleep disturbance.

To test dose-response relationships, we determined the number of daily defined doses (DDDs) of Z-drugs and benzodiazepines at each prescription. The DDD is the assumed average maintenance dose per day for a drug based on its main indication in adults. We used DDD values from the World Health Organization’s Collaborating Centre for Drug Statistics Methodology (www.whocc.no/atc_ddd_index), where the DDDs for zopiclone, zolpidem, and zaleplon are 7.5 mg, 10 mg, and 10 mg per day, respectively. The British National Formulary recommends these daily doses for adults with insomnia, but to halve them for elderly patients. Missing dosing frequency data was assumed once daily, except diazepam where we applied the most common frequency for the product and quantity prescribed among the complete prescription data.

### Outcomes

The selected outcomes were identified from previous studies or were priorities identified by our Patient and Public Involvement (PPI) group members or by an advisory group of healthcare professionals established to support this project. The main outcomes were, in order of importance: (1) incident (a) fracture in any location, (b) hip fracture, and (c) forearm/wrist/hand fracture; (2) incident fall; (3) mortality; (4) acute bacterial infection; (5) ischaemic stroke/transient ischaemic attack; and (6) venous thromboembolism. These were identified via first mention of a relevant Read code in CPRD or ICD-10 code in HES or ONS (codes in Additional file 3). We also examined further healthcare utilisation outcomes: (7) number of (a) hospital admissions and (b) GP consultations and (8) new prescription of (a) antipsychotics, (b) antidepressants, and (c) antibiotics.

### Covariates

We considered as potential confounders variables suspected to be linked to dementia, sleep disturbance, benzodiazepine or Z-drug use, or the outcomes examined. They were measured on the index date and covered domains of demographics, health behaviours, dementia subtype, proxies for dementia severity, proxies for sleep disturbance severity, comorbidities, recent medical history (e.g. GP consultations, hospital admissions, falls, fractures, infections, immunisations, body mass index [BMI], systolic blood pressure), and concurrent prescriptions (details in Additional file 4).

### Statistical analysis

The primary analysis estimated the association between new prescription of sleep disturbance medication and incidence of each outcome, compared to other groups. We followed patients until the earliest of death, leaving the GP practice, last data extraction, new sedative-hypnotic or antipsychotic prescription, 2 years post-index date, or 31 March 2016. Z-drug and benzodiazepine new users were also censored 90 days following their last Z-drug/benzodiazepine prescription. Matched patients were additionally censored at the censoring date of their corresponding case. Specific exclusion criteria applied at the index date to reduce the chance of repeated coding of the same event are described in Additional file 5 table S1.

Cox proportional hazards regression was used for binary outcomes. We used robust standard errors to account for the correlation due to repeat measurements in some patients [27]. The proportional hazards assumption was checked using Schoenfeld residuals [28]. Negative binomial regression was used to model the number of hospital admissions and GP consultations. Estimates were adjusted for age and sex and all covariates in Additional file 4. Age, BMI, systolic blood pressure, duration since dementia diagnosis, index date, and number of prior GP consultations were modelled using restricted cubic splines (with five knots) to allow non-linear effects [29]. We included an interaction between sex and BMI, due to known sex differences in the relationship between BMI and fracture risk [30]. Absolute risk differences of adverse events and numbers needed to harm (NNH) were estimated using standard formulae for time to event analysis [31].

We examined the average daily Z-drug dose over follow-up, but in post hoc secondary analysis, to reflect changes in dose, we examined time-varying daily DDDs. In the sensitivity analysis, we excluded those with record of > 6 h sleep per night from the sleep-disturbance comparator group (see validation study in Additional file 2 for more detail). This was to increase the chance that the sleep-disturbance group had more comparable insomnia to the Z-drug group. Finally, in the comparison of Z-drug to benzodiazepine new users, we restricted to benzodiazepines likely prescribed for sleep disturbance (loprazolam, lormetazepam, nitrazepam, temazepam, or other benzodiazepines with dosing instructions to take only at night or with a concurrent record of sleep disturbance).

Multiple imputation by chained equations was used to impute missing values of BMI, smoking, alcohol use, residence, ethnicity, and blood pressure (see Additional file 4 for details) [32]. To account for multiple outcomes tested, we used the Benjamini-Hochberg procedure to control the false discovery rate at < 5% for each analysis [33]. Stata version 15.1 was used throughout.

## Results

There were 51,117 eligible dementia patients with ≥ 12-month data history in the linked CPRD-HES database (Additional file 5 figure S1). Of these, 3532 and 5172 patients were newly prescribed Z-drugs or benzodiazepines, respectively, and met our inclusion criteria. Z-drug and benzodiazepine new users were matched to 10,214 and 15,174 non-users, respectively. Finally, 1833 dementia patients had recorded sleep disturbance, but without prescription of sedative-hypnotics.

### Patient characteristics

The mean (SD) age of patients at index date was 83 (7.7) years, and 16,802 (62%) were women. Patients were registered with their GP for a median (IQR) of 19 (11–32) years and diagnosed with dementia for a median (IQR) of 12 (4–25) months.

The patient cohorts were similar across measured characteristics (Table 1 with missing data described in Additional file 5 table S2). Recent hospital admissions were more likely among Z-drug users. Concurrent antipsychotics and antidepressants and previous benzodiazepine or Z-drug use was more likely among benzodiazepine and Z-drug users. Benzodiazepine users more frequently lived in care homes and had agitation/psychosis and anxiety and dementia for longer. Those with sleep disturbance (no sedative-hypnotics) were more likely from a deprived neighbourhood, consume alcohol, and have urinary incontinence and insomnia history before dementia. Finally, the non-users with proximal GP consultation had more recent GP consultations and fewer recent falls and fractures.

**Table 1:** Characteristics of patients with dementia prescribed Z-drugs and benzodiazepines and comparison cohorts

	Z-drug (***n*** = 3532)		Sleep disturbance, no sedative-hypnotic (***n*** = 1833)		No Z-drug, proximal GP consultation (***n*** = 10,214)		Benzodiazepine (***n*** = 5172)		No benzodiazepine, proximal GP consultation (***n*** = 15,174)	
Characteristic	***n***	%	***n***	%	***n***	%	***n***	%	***n***	%
Women	2087	59%	1145	62%	6074	59%	3222	62%	9491	63%
Age, years^a^	82.9	7.7	83.1	7.1	83.1	7.5	82.5	7.8	82.6	7.8
White ethnicity^b^	3072	87%	1539	84%	8816	86%	4470	86%	13,042	86%
Care home^b^	892	25%	434	24%	2108	21%	1509	29%	2979	20%
Lives alone^b^	760	22%	545	30%	2848	28%	993	19%	4785	32%
GP practice area IMD quintile^a^	3.2	1.4	3.4	1.4	3.1	1.4	3.2	1.4	3.1	1.4
Current smoker^b^	312	9%	142	8%	722	7%	355	7%	1124	7%
Ex-smoker^b^	765	22%	428	23%	2226	22%	1163	22%	3322	22%
Alcohol drinker^b^	715	20%	489	27%	2142	21%	1127	22%	3297	22%
Body mass index^a,b^	24.9	4.9	24.5	4.6	24.9	4.8	24.5	4.8	24.8	4.9
Systolic blood pressure^a,b^	133.5	19.0	133.9	18.8	134.3	19.1	133.6	18.9	134.5	18.8
**Dementia**										
Months since dementia diagnosis^c^	11.4	3.6–26.2	11.0	3.8–24.1	13.5	5.2–27.4	15.6	5.1–32.4	10.3	3.7–22.0
Dementia subtype										
Alzheimer’s disease	1355	38%	746	41%	4224	41%	2140	41%	6612	44%
Vascular dementia	940	27%	487	27%	2773	27%	1375	27%	4046	27%
Other/mixed dementia	409	12%	192	10%	1013	10%	600	12%	1377	9%
Unspecified dementia	828	23%	403	22%	2184	21%	1057	20%	3139	21%
Agitation/psychosis history	619	18%	409	22%	1261	12%	1363	26%	1341	9%
End of life care	197	6%	77	4%	564	6%	416	8%	726	5%
**Sleep disturbance**										
Sleep disturbance pre-dementia	825	23%	615	34%	1982	19%	1064	21%	2918	19%
History of benzodiazepine use	869	25%	222	12%	1376	13%	982	19%	2009	13%
History of Z-drug use	304	9%	113	6%	597	6%	481	9%	852	6%
**Medical history in the past year**										
Falls	1003	28%	542	30%	2165	21%	1241	24%	3359	22%
Fractures	360	10%	131	7%	657	6%	409	8%	1071	7%
Dizziness/unsteadiness	214	6%	127	7%	652	6%	294	6%	817	5%
Faints/syncope	184	5%	120	7%	533	5%	357	7%	712	5%
Urinary tract infection/acute LRTI	928	26%	423	23%	2054	20%	1242	24%	3114	21%
Influenza vaccination	2480	70%	1340	73%	7747	76%	3739	72%	11,350	75%
Pneumonia vaccination	166	5%	103	6%	506	5%	234	5%	773	5%
Physician consultations^a^	12.5	10.3	11.2	9.4	14.9	12.7	12.9	10.7	13.7	12.0
Hospital admissions^a^	1.3	3.0	1.0	2.2	0.9	1.6	1.1	1.9	0.9	2.3
**Comorbidities**										
Depression	894	25%	493	27%	2633	26%	1512	29%	3723	25%
Depression symptoms	692	20%	368	20%	1898	19%	1175	23%	2804	18%
Anxiety	576	16%	311	17%	1648	16%	1204	23%	2451	16%
Anxiety symptoms	446	13%	261	14%	1181	12%	877	17%	1751	12%
Parkinson’s disease	208	6%	109	6%	530	5%	290	6%	723	5%
Urinary incontinence	520	15%	465	25%	1640	16%	841	16%	2273	15%
Benign prostatic hyperplasia	361	10%	175	10%	1075	11%	487	9%	1474	10%
Asthma	366	10%	165	9%	1056	10%	517	10%	1590	10%
Cancer	743	21%	313	17%	2114	21%	1002	19%	3074	20%
COPD	266	8%	147	8%	782	8%	369	7%	1209	8%
Osteoporosis	417	12%	215	12%	1224	12%	581	11%	1885	12%
Other muscleroskeletal conditions	448	13%	248	14%	1395	14%	664	13%	2036	13%
Osteoarthritis/rheumatoid arthritis	1426	40%	756	41%	3980	39%	2063	40%	5906	39%
Other joint conditions	2901	82%	1537	84%	8463	83%	4293	83%	12,615	83%
Headache/migraine	720	20%	363	20%	2020	20%	1101	21%	2955	19%
Back/neck pain	1959	55%	1009	55%	5640	55%	2910	56%	8380	55%
Age-related macular degeneration	189	5%	115	6%	648	6%	348	7%	923	6%
Cataract	992	28%	547	30%	2940	29%	1400	27%	4284	28%
Glaucoma	356	10%	186	10%	1011	10%	445	9%	1459	10%
Retinal disorder	295	8%	138	8%	944	9%	415	8%	1390	9%
Diabetes	531	15%	245	13%	1712	17%	718	14%	2483	16%
Hyperlipidaemia	470	13%	269	15%	1443	14%	754	15%	2152	14%
Hypertension	1822	52%	1006	55%	5736	56%	2762	53%	8521	56%
Stroke/transient ischaemic attack	783	22%	397	22%	2177	21%	1091	21%	3022	20%
Myocardial infarction	310	9%	172	9%	909	9%	433	8%	1307	9%
Heart failure	314	9%	182	10%	988	10%	444	9%	1350	9%
Atrial fibrillation	528	15%	260	14%	1666	16%	775	15%	2365	16%
Angina	532	15%	308	17%	1646	16%	811	16%	2263	15%
Venous thromboembolism	236	7%	123	7%	737	7%	341	7%	1029	7%
**Prescriptions in the last 90 days**										
Anticholinesterase/memantine	850	24%	385	21%	2417	24%	1237	24%	3662	24%
Antipsychotic	811	23%	371	20%	1586	16%	1125	22%	851	6%
SSRI antidepressant	763	22%	352	19%	1855	18%	1174	23%	2616	17%
Tricyclic antidepressant	363	10%	192	10%	835	8%	596	12%	1748	12%
Other antidepressant	294	8%	153	8%	575	6%	483	9%	771	5%
Antiepileptic	241	7%	110	6%	610	6%	424	8%	825	5%
Analgesic	1730	49%	808	44%	4107	40%	2338	45%	5805	38%
Inhaled corticosteroid	191	5%	96	5%	507	5%	196	4%	742	5%
Lipid regulating medication	1217	34%	630	34%	3492	34%	1644	32%	5324	35%
Diuretic	1188	34%	633	35%	3322	33%	1456	28%	4779	31%
Beta blocker	645	18%	328	18%	1907	19%	850	16%	2771	18%
ACE inhibitor	709	20%	379	21%	2222	22%	991	19%	3357	22%
Angiotensin II receptor antagonist	235	7%	124	7%	724	7%	330	6%	1123	7%
Calcium channel blocker	643	18%	339	18%	1947	19%	849	16%	2980	20%
Anticoagulant	204	6%	103	6%	739	7%	295	6%	1056	7%
Antiplatelet	1603	45%	878	48%	4390	43%	2171	42%	6439	42%
Cardiac glycoside	281	8%	155	8%	768	8%	345	7%	1032	7%
NSAID	352	10%	156	9%	815	8%	468	9%	1206	8%
Bisphosphonate	375	11%	194	11%	1074	11%	477	9%	1618	11%
Calcium/vitamin D	677	19%	377	21%	1854	18%	888	17%	2754	18%
Antibiotic (in last 30 days)	1098	31%	567	31%	2590	25%	1370	26%	3640	24%

*Abbreviations*: *ACE* angiotensin-converting enzyme, *COPD* chronic obstructive pulmonary disease, *IMD* Index of Multiple Deprivation, *GP* general practitioner, *LRTI* lower respiratory tract infection, *NSAID* nonsteroidal anti-inflammatory drug, *SSRI* selective serotonin reuptake inhibitor

^a^Mean (standard deviation)

^b^Characteristic contains missing data as described in Additional file 5 table S2

^c^Median (inter-quartile range)

Of 3532 patients prescribed Z-drugs, 3358 (95%) were prescribed zopiclone, with 2801 (83%) prescribed 3.75 mg daily on the index date. For 598 (17%), the prescription instructions were ‘pro re nata’ (PRN/as needed). Of 5172 patients prescribed benzodiazepines, the most common were diazepam (*n* = 2077, 40%), lorazepam (*n* = 1669, 32%), and temazepam (*n* = 1168, 23%). Patients were followed up for a median (IQR) of 3.5 (3.0–10.3) months and mainly censored due to no further Z-drug or benzodiazepine prescriptions. See Additional file 5 table S3 for initial and follow-up doses.

### Falls and fractures

We estimated HRs (95% CI) of 1.32 (0.99–1.75) and 1.34 (1.08–1.67) for Z-drugs and fracture compared to sleep disturbance (without sedative-hypnotics) and non-use with proximal GP consultation, respectively (Table 2). For hip fracture, the HRs (95% CI) were 1.38 (0.92–2.06) and 1.59 (1.15–2.19) for Z-drugs compared to sleep disturbance and non-use with proximal GP consultation, respectively. Z-drug use was associated with increased falls compared to non-use with proximal GP consultation (HR 1.43, 95% CI 1.26–1.62), but not compared to sleep disturbance (HR 1.02, 95% CI 0.87–1.21). New benzodiazepine use was associated with increased fractures and falls compared to non-use with proximal GP consultation, but the HR (95% CI) for hip fractures was 1.17 (0.87–1.57) (Additional file 5 table S4). There were no large differences in fall and fracture rates between new Z-drug and benzodiazepine users (Table 2).

**Table 2:** Adjusted hazard ratios for new Z-drug prescription and adverse events for people with dementia

Outcome		Comparator		
	Z-drug (*n* = 3532)	Sleep disturbance, no sedative-hypnotic (*n* = 1833)	No Z-drug, proximal GP consultation (*n* = 10,214)	Benzodiazepine (*n* = 5172)
Fracture				
Incidence rate per 100PY (events)	11.4 (164)	7.6 (130)	8.4 (269)	12.5 (223)
Age, sex-adjusted HR (95% CI)	NA	1.39 (1.08–1.78)	1.40 (1.14–1.70)	0.93 (0.76–1.14)
Fully adjusted HR (95% CI)^a^	NA	1.32 (0.99–1.75)	1.34 (1.08–1.67)^b^	0.99 (0.80–1.23)
Hip fracture				
Incidence rate per 100PY (events)	5.7 (84)	3.4 (60)	3.5 (115)	5.1 (94)
Age, sex-adjusted HR (95% CI)	NA	1.53 (1.07–2.18)	1.64 (1.23–2.19)	1.11 (0.82–1.49)
Fully adjusted HR (95% CI)^a^	NA	1.38 (0.92–2.06)	1.59 (1.15–2.19)^b^	1.10 (0.87–1.65)
Forearm/wrist/hand fracture				
Incidence rate per 100PY (events)	2.0 (29)	1.1 (20)	1.5 (48)	2.5 (46)
Age, sex-adjusted HR (95% CI)	NA	1.80 (0.95–3.41)	1.35 (0.85–2.14)	0.81 (0.51–1.28)
Fully adjusted HR (95% CI)^a^	NA	1.44 (0.60–3.47)	1.33 (0.77–2.31)	1.00 (0.59–1.70)
Fall				
Incidence rate per 100PY (events)	37.1 (473)	27.3 (384)	25.8 (767)	35.8 (585)
Age, sex-adjusted HR (95% CI)	NA	1.12 (0.97–1.29)	1.52 (1.36–1.71)	1.06 (0.94–1.20)
Fully adjusted HR (95% CI)^a^	NA	1.02 (0.87–1.21)	1.43 (1.26–1.62)^b^	1.08 (0.95–1.22)
Mortality				
Incidence rate per 100PY (events)	28.4 (436)	16.7 (301)	24.1 (799)	39.0 (736)
Age, sex-adjusted HR (95% CI)	NA	1.51 (1.29–1.77)	1.20 (1.06–1.34)	0.72 (0.64–0.81)
Fully adjusted HR (95% CI)^a^	NA	1.38 (1.14–1.66)^b^	1.08 (0.94–1.23)	0.73 (0.64–0.83)^b^
Acute bacterial infection				
Incidence rate per 100PY (events)	47.8 (416)	43.6 (220)	40.6 (1325)	57.8 (371)
Age, sex-adjusted HR (95% CI)	NA	1.13 (0.94–1.37)	1.24 (1.09–1.42)	1.01 (0.86–1.18)
Fully adjusted HR (95% CI)^a^	NA	1.02 (0.82–1.27)	1.13 (0.98–1.31)	0.92 (0.78–1.10)
Ischaemic stroke/transient ischaemic attack				
Incidence rate per 100PY (events)	6.2 (93)	4.4 (77)	5.5 (178)	6.0 (110)
Age, sex-adjusted HR (95% CI)	NA	1.32 (0.95–1.83)	1.20 (0.94–1.54)	1.03 (0.78–1.36)
Fully adjusted HR (95% CI)^a^	NA	1.35 (0.90–2.04)	1.14 (0.86–1.50)	1.05 (0.78–1.43)
Venous thromboembolism				
Incidence rate per 100PY (events)	1.5 (22)	1.3 (21)	1.4 (43)	2.5 (47)
Age, sex-adjusted HR (95% CI)	NA	1.64 (0.97–2.79)	1.14 (0.74–1.76)	0.79 (0.50–1.25)
Fully adjusted HR (95% CI)^a^	NA	1.65 (0.74–3.69)^c^	1.12 (0.67–1.85)	0.82 (0.50–1.34)

*Abbreviations*: *HR* hazard ratio, *CI* confidence interval, *GP* general practitioner, *PY* person-years

^a^Adjusted for all covariates listed in Table 1

^b^Fully adjusted HR remaining statistically significant after controlling the false discovery rate to < 5% (based on 13 outcomes)

^c^Not adjusted for antiepileptics, antiplatelet drugs, pneumonia vaccine, and anxiety symptoms due to model instability

There was evidence of differing associations with the outcomes according to the prescribed daily dose of Z-drugs or benzodiazepines (Table 3 and Additional file 5 table S5). Compared to sleep disturbance without sedative-hypnotics, the adjusted HRs (95% CI) for fractures, hip fractures, and falls for Z-drug prescriptions equivalent to ≥ 7.5 mg zopiclone daily were 1.67 (1.13–2.46), 1.96 (1.16–3.31), and 1.33 (1.06–1.66), respectively. The adjusted HRs (95% CI) for Z-drug prescriptions equivalent to ≤ 3.75 mg zopiclone daily and fractures, hip fractures, and falls were 1.22 (0.90–1.66), 1.21 (0.78–1.90), and 0.95 (0.80–1.13), respectively. Similar associations were observed when compared to non-users with proximal GP consultation.

**Table 3:** Adjusted hazard ratios for Z-drug prescription and adverse events for people with dementia according to prescribed daily defined dose of Z-drugs

Outcome and daily defined dose prescribed^a^	No. events in the Z-drug cohort	Sleep disturbance and no sedative-hypnotic (*n* = 1833)		No Z-drug, proximal GP consultation (*n* = 10,214)	
		Age, sex adjusted	Fully adjusted^b^	Age, sex adjusted	Fully adjusted^b^
Fracture					
≤ 0.5	117	1.30 (0.99–1.70)	1.22 (0.90–1.66)	1.33 (1.07–1.65)	1.28 (1.01–1.63)
0.6–0.9	5	1.28 (0.51–3.20)	1.06 (0.39–2.89)	1.24 (0.50–3.05)	1.08 (0.42–2.76)
≥ 1	42	1.70 (1.19–2.42)	1.67 (1.13–2.46)^c^	1.66 (1.18–2.34)	1.58 (1.09–2.28)^c^
Hip fracture					
≤ 0.5	55	1.30 (0.88–1.93)	1.21 (0.78–1.90)	1.42 (1.03–1.97)	1.43 (1.00–2.06)
0.6–0.9	< 5	1.10 (0.26–4.67)	0.79 (0.15–4.11)	1.27 (0.31–5.22)	1.05 (0.24–4.64)
≥ 1	27	2.30 (1.45–3.65)	1.96 (1.16–3.31)^c^	2.50 (1.61–3.89)	2.36 (1.44–3.87)^c^
Forearm fracture					
≤ 0.5	20	1.66 (0.81–3.40)	1.22 (0.48–3.12)	1.28 (0.77–2.15)	1.29 (0.73–2.27)
≥ 0.6	9	2.18 (0.98–4.85)	1.91 (0.67–5.47)	1.53 (0.72–3.24)	1.42 (0.59–3.38)
Fall					
≤ 0.5	335	1.05 (0.90–1.22)	0.95 (0.80–1.13)	1.43 (1.26–1.63)	1.35 (1.17–1.56)^c^
0.6–0.9	14	0.84 (0.49–1.44)	0.73 (0.41–1.29)	1.19 (0.70–2.02)	1.07 (0.61–1.87)
≥ 1	124	1.42 (1.15–1.74)	1.33 (1.06–1.66)^c^	1.92 (1.58–2.35)	1.81 (1.46–2.34)^c^
Mortality					
≤ 0.5	321	1.49 (1.26–1.77)	1.38 (1.14–1.68)^c^	1.18 (1.03–1.34)	1.07 (0.93–1.24)
0.6–0.9	16	1.56 (0.95–2.56)	1.60 (0.96–2.70)	1.25 (0.77–2.02)	1.22 (0.75–1.99)
≥ 1	99	1.56 (1.24–1.96)	1.33 (1.03–1.71)	1.26 (1.02–1.55)	1.06 (0.85–1.33)
Acute bacterial infection					
≤ 0.5	297	1.15 (0.98–1.36)	1.00 (0.83–1.20)	1.37 (1.20–1.57)	1.23 (1.06–1.43)^c^
0.6–0.9	13	0.98 (0.56–1.72)	0.86 (0.47–1.57)	1.20 (0.69–2.08)	1.12 (0.63–1.99)
≥ 1	106	1.40 (1.12–1.76)	1.25 (0.98–1.60)	1.64 (1.32–2.04)	1.52 (1.21–1.91)^c^
Ischaemic stroke/transient ischaemic attack					
≤ 0.5	55	1.04 (0.72–1.51)	1.12 (0.71–1.75)	0.96 (0.71–1.30)	0.95 (0.68–1.31)
0.6–0.9	5	1.97 (0.78–4.96)	1.98 (0.74–5.28)	1.74 (0.72–4.19)	1.71 (0.74–3.98)
≥ 1	33	2.07 (1.36–3.15)	1.88 (1.14–3.10)^c^	1.90 (1.30–2.79)	1.61 (1.08–2.42)
Venous thromboembolism					
≤ 0.5	26	2.01 (1.16–3.49)	2.00 (0.90–4.47)	1.35 (0.85–2.13)	1.26 (0.74–2.12)
≥ 0.6	< 5	0.78 (0.27–2.25)	0.85 (0.21–3.39)	0.56 (0.21–1.54)	0.66 (0.22–1.95)

*Abbreviations*: *HR* hazard ratio, *CI* confidence interval, *GP* general practitioner

^a^The reference group for all comparisons is no Z-drug prescription. Most patients assigned to the ‘0.6–0.9 DDD’ Z-drug group were prescribed 3.75 mg zopiclone with instructions similar to ‘TAKE ONE OR TWO AT NIGHT’

^b^Adjusted for all covariates listed in Table 1

^c^Fully adjusted HR remaining statistically significant after controlling the false discovery rate to < 5% (based on 37 tests; 11 outcomes with three dose categories and two outcomes with two dose categories)

#### Absolute risks

The use of zopiclone at ≥ 7.5 mg or equivalent is associated with absolute annual risks of fracture of 12.4% (compared to 7.6% in the sleep disturbance cohort). For hip fracture, the corresponding figures are 6.6% annual risk associated with zopiclone at ≥ 7.5 mg or equivalent compared to 3.4%. This is equivalent to NNH of 21 and 32, and extra cases per 1000 treated of 48 and 32 for fractures and hip fractures, respectively.

### Mortality, infection, and cardiovascular outcomes

Although Z-drug use was associated with greater mortality compared to those with sleep disturbance (HR 1.38, 95% CI 1.14–1.66), there was no strong evidence of excess risk compared to non-users with a proximal GP consultation (HR 1.08, 95% CI 0.94–1.23) (Table 2). Further, the associations with mortality seemed unrelated to dose (Table 3). Z-drug prescription was associated with less mortality than benzodiazepines (HR 0.73, 95% CI 0.64–0.83).

We did not detect any strong associations between new Z-drug prescription and greater infection or venous thromboembolism rates, compared either to the non-users or to the benzodiazepine users.

When examining new Z-drug prescription overall, we did not detect strong associations with incident stroke rates (HR 1.14 [95% CI 0.86–1.50], compared to non-users with proximal GP consultation). However, higher dose (≥ 7.5 mg zopiclone or equivalent) Z-drugs were associated with more ischaemic strokes (HR 1.88 [95% CI 1.14–3.10] and 1.90 [1.30–2.79] compared to sleep disturbance and non-users with proximal GP consultation). The association for higher dose Z-drugs appeared greater than that for higher dose (> 5 mg diazepam or equivalent) benzodiazepine use, with a HR (95% CI) for higher dose benzodiazepine and stroke of 1.37 (0.91–2.08) compared to non-use with proximal GP consultation (Additional file 5 table S5).

#### Absolute risks

The use of zopiclone at ≥ 7.5 mg or equivalent is associated with absolute annual risks of stroke of 8.1% (compared to 4.4% in the sleep disturbance cohort). This is equivalent to an NNH of 27 and 37 extra cases per 1000 treated.

### Additional medication and healthcare utilisation

The adjusted rate ratios (95% CI) for hospital visits for Z-drug users were 1.26 (1.13–1.40), compared to sleep disturbance, and 1.17 (1.07–1.27), compared to non-use with proximal GP consultation. The rates between Z-drugs and benzodiazepines were similar. For GP consultations, the corresponding rate ratios were 1.17 (1.12–1.23) and 1.07 (1.04–1.11), respectively. However, when analysed by time-varying prescribed dose, more frequent hospital admissions and GP consultations were generally only observed for higher dose Z-drugs (Table 5) and higher dose benzodiazepines (Additional file 5 table S6).

Z-drug users were more likely prescribed a new antipsychotic (HR 2.37, 95% CI 1.84–3.04) or antidepressant (HR 2.32, 95% CI 1.65–3.25) during follow-up compared to non-users with sleep disturbance (Table 4). There was a small increase in antibiotic prescribing subsequent to new Z-drug prescription compared to non-users with proximal GP consultation (HR 1.19, 95% CI 1.08–1.30). Rates of new prescribing were generally similar post-Z-drug prescription to post-benzodiazepine prescription and were greater with increasing Z-drug dose (Table 5).

**Table 4:** Adjusted hazard and rate ratios for new Z-drug prescription and new prescriptions, GP consultations, and hospital admissions for people with dementia

Outcome		Comparator		
	Z-drug (*n* = 3532)	Sleep disturbance, no sedative-hypnotic (*n* = 1833)	No Z-drug, proximal GP consultation (*n* = 10,214)	Benzodiazepine (*n* = 5172)
Number of hospital admissions				
Rate per 100PY (events)	126.7 (1944)	93.0 (1671)	107.7 (3563)	129.4 (2441)
Age, sex-adjusted RR (95% CI)	NA	1.34 (1.20–1.49)	1.26 (1.15–1.39)	0.98 (0.89–1.09)
Fully adjusted RR (95% CI)^a^	NA	1.26 (1.13–1.40)^b^	1.17 (1.07–1.27)^b^	0.92 (0.84–1.01)
Number of GP consultations				
Rate per 100PY (events)	1387.8 (21292)	1124.6 (20209)	1511.7 (50021)	1502.6 (28335)
Age, sex-adjusted RR (95% CI)	NA	1.29 (1.22–1.37)	0.97 (0.93–1.01)	0.94 (0.90–0.98)
Fully adjusted RR (95% CI)^a^	NA	1.17 (1.12–1.23)^b^	1.07 (1.04–1.11)^b^	0.96 (0.93–1.00)
New antipsychotic prescription				
Incidence rate per 100PY (events)	38.7 (331)	10.3 (130)	15.2 (227)	75.4 (532)
Age, sex-adjusted HR (95% CI)	NA	2.53 (2.03–3.14)	3.68 (3.10–4.37)	0.79 (0.68–0.90)
Fully adjusted HR (95% CI)^a^	NA	2.37 (1.84–3.04)^b^	3.85 (3.18–4.65)^b^	0.86 (0.74–0.99)
New antidepressant prescription				
Incidence rate per 100PY (events)	23.8 (172)	8.2 (82)	10.3 (199)	30.3 (242)
Age, sex-adjusted HR (95% CI)	NA	2.24 (1.70–2.94)	2.52 (2.05–3.10)	0.86 (0.71–1.05)
Fully adjusted HR (95% CI)^a^	NA	2.32 (1.65–3.25)^b^	2.65 (2.09–3.37)^b^	0.88 (0.71–1.10)
New antibiotic prescription				
Incidence rate per 100PY (events)	109.1 (791)	61.2 (517)	89.5 (1738)	111.0 (1096)
Age, sex-adjusted HR (95% CI)	NA	1.46 (1.30–1.65)	1.27 (1.17–1.38)	1.01 (0.92–1.10)
Fully adjusted HR (95% CI)^a^	NA	1.34 (1.17–1.52)^b^	1.19 (1.08–1.30)^b^	1.05 (0.95–1.15)

*Abbreviations*: *HR* hazard ratio, *CI* confidence interval, *PY* person-years, *RR* rate ratio

^a^Adjusted for all covariates listed in Table 1

^b^Fully adjusted HR remaining statistically significant after controlling the false discovery rate to < 5% (based on 13 outcomes)

**Table 5:** Adjusted hazard ratios for Z-drug prescription and new prescriptions, GP consultations, and hospital admissions for people with dementia according to prescribed daily defined doses (DDDs) of Z-drugs

Outcome and daily defined dose prescribed^a^	No. events in the Z-drug cohort	No sedative-hypnotic, sleep disturbance (*n* = 1833)		No Z-drug, proximal GP consultation (*n* = 10,214)	
		Age, sex adjusted	Fully adjusted^b^	Age, sex adjusted	Fully adjusted^b^
Number of hospital admissions^c^					
≤ 0.5	1403	1.24 (1.10–1.39)	1.18 (1.05–1.32)	1.19 (1.07–1.31)	1.10 (1.00–1.20)
0.6–0.9	63	1.06 (0.76–1.49)	1.22 (0.88–1.69)	1.03 (0.74–1.43)	1.10 (0.80–1.51)
≥ 1	472	1.35 (1.15–1.58)	1.29 (1.10–1.50)^d^	1.32 (1.15–1.52)	1.22 (1.06–1.39)^d^
Number of GP consultations^c^					
≤ 0.5	9230	1.21 (1.14–1.29)	1.10 (1.05–1.16)^d^	0.89 (0.85–0.94)	1.00 (0.96–1.04)
0.6–0.9	690	1.12 (0.96–1.32)	1.10 (0.95–1.26)	0.83 (0.71–0.97)	1.02 (0.88–1.17)
≥ 1	4823	1.23 (1.12–1.34)	1.16 (1.08–1.25)^d^	0.91 (0.84–0.98)	1.06 (0.99–1.13)
Incident antipsychotic prescription					
≤ 0.5	214	2.08 (1.64–2.62)	2.00 (1.54–2.61)^d^	3.09 (2.56–3.74)	3.31 (2.69–4.07)^d^
0.6–0.9	18	3.35 (2.04–5.50)	2.75 (1.57–4.81)^d^	5.15 (3.19–8.32)	4.82 (2.81–8.25)^d^
≥ 1	99	4.05 (3.10–5.31)	3.56 (2.61–4.85)^d^	5.81 (4.55–7.42)	5.82 (4.44–7.63)^d^
Incident antidepressant prescription					
≤ 0.5	124	1.25 (1.14–1.37)	1.16 (1.05–1.28)^d^	2.47 (1.91–3.19)	2.39 (1.90–2.99)^d^
0.6–0.9	8	1.20 (0.83–1.73)	1.13 (0.76–1.68)	2.67 (1.19–5.99)	3.00 (1.49–6.01)
≥ 1	40	1.37 (1.17–1.61)	1.31 (1.11–1.54)^d^	3.47 (2.32–5.18)	2.93 (2.04–4.19)^d^
Incident antibiotic prescription					
≤ 0.5	583	1.43 (1.27–1.63)	1.30 (1.13–1.49)^d^	1.25 (1.14–1.37)	1.16 (1.05–1.28)^d^
0.6–0.9	27	1.36 (0.93–2.00)	1.20 (0.79–1.81)	1.20 (0.83–1.73)	1.13 (0.76–1.68)
≥ 1	181	1.57 (1.32–1.88)	1.46 (1.21–1.76)^d^	1.37 (1.17–1.61)	1.31 (1.11–1.54)^d^

*Abbreviations*: *HR* hazard ratio, *CI* confidence interval, *GP* general practitioner

^a^The reference group for all comparisons is no Z-drug prescription. Most patients assigned to the ‘0.6–0.9 DDD’ Z-drug group were prescribed 3.75 mg zopiclone with instructions similar to ‘TAKE ONE OR TWO AT NIGHT’

^b^Adjusted for all covariates listed in Table 1

^c^Estimates provided are rate ratios (95% confidence intervals)

^d^Fully adjusted HR remaining statistically significant after controlling the false discovery rate to < 5% (based on 37 tests; 11 outcomes with three dose categories and two outcomes with two dose categories)

### Additional analyses

Similar associations to those for time-varying prescribed dose were observed when analysing the (non-time-varying) average number of doses prescribed over the exposure period (Additional file 5 table S7-S8). In the sensitivity analysis using a comparator of those with sleep disturbance and no associated mention of > 6 h sleep, associations for Z-drug use were generally slightly reduced (Additional file 5 tables S9-S10). Finally, associations for Z-drug use compared to the 1601 patients prescribed benzodiazepines for sleep disturbance were very similar to when comparing to any benzodiazepine (Additional file 5, table S11).

## Discussion

We found evidence of increased risks of falls, fractures, and ischaemic stroke in people with dementia prescribed Z-drugs at higher doses. The associations observed were similar or greater in magnitude to those for higher dose benzodiazepine prescription. One in six Z-drug prescriptions were commenced at higher doses of equivalent to 7.5 mg zopiclone daily or greater. We did not detect any consistent or clinically significant increased risks of mortality, infection, or venous thromboembolism with Z-drug use. PlwD prescribed higher dose Z-drugs were also more likely to be admitted to hospital, visit their GP, and be further prescribed antipsychotics, antidepressants, and antibiotics.

We designed the study to minimise possible sources of bias [34]. Although we were unable to measure dementia severity, we adjusted for the duration since the dementia diagnosis, prescription of dementia medications and antipsychotics, history of agitation/psychosis, and end of life care. However, there may be residual confounding by dementia severity for some comparisons. Although underlying severity was unclear, the mortality results suggest that, compared to Z-drug users, the sleep disturbance group not prescribed sedatives may have less severe dementia, however that the non-users with proximal GP consultation had comparable dementia severity. Residual confounding by sleep disturbance severity is also a possibility. Sleep disturbance was challenging to identify within the electronic primary care record as highlighted in our validation study, where only 42% of our selected ‘sleep disturbance’ patients had sleep disturbance confirmed by their GP practice. This could be partly due to the sometimes contradictory ‘sleep pattern’ records or that the sleep disturbance recorded was transient or due to other causes, such as urinary incontinence or alcohol abuse. The comparable fall risk in the Z-drug and ‘sleep disturbance’ group could be due to urinary incontinence and alcohol use being more common in the ‘sleep disturbance’ group. The coding of ‘sleep disturbance’ was often vague and may represent conditions other than insomnia. It may be that some of the ‘sleep disturbance and no sedative’ group had milder cases of sleep disturbance than those prescribed Z-drugs. Performing a sensitivity analysis on the sleep disturbance definition reduced our effects slightly. Residual confounding by severity of insomnia or dementia could also affect the associations with higher Z-drug doses. We also had no data on genetic information and environmental factors, which may influence fall risk in people living with dementia [35]. There was likely some small residual confounding due to new admission to a care home; however, we were unable to accurately ascertain the admission date to control for this.

Our study was strengthened by additional comparisons to non-users with a proximal GP visit and new benzodiazepine users. As benzodiazepines are also prescribed for anxiety and behavioural disturbances of dementia, there may be residual confounding by dementia and sleep disturbance severity in the comparison between Z-drug and benzodiazepine users. However, when instead restricting to benzodiazepines likely prescribed for sleep disturbance, our findings were very similar. Dosing instructions were often missing for benzodiazepines, and although we made plausible assumptions based on the complete prescriptions, some misclassification of exposures is possible. Recording of prescriptions issued in primary care is accurate; however, we lacked data on medications prescribed in secondary care or obtained elsewhere. Medication adherence is unknown; therefore, the Z-drug effects may be underestimated if many patients prescribed Z-drugs had not taken them. Studies report high positive predictive values for patients coded with our study outcomes in UK primary care data [36–41]. Potential under-reporting in CPRD was improved through linkage to HES and ONS. However, we likely underestimated forearm fracture incidence as many do not require hospital admission. Similarly, GP records of falls may under-represent all falls that occur in the older population, but more accurately represent ‘injurious falls requiring medical attention’ [42]. Our study was strengthened by using a new-user design and careful selection and follow-up of patients taking Z-drugs alone and not concurrent with other sedative-hypnotics [43, 44]. Our findings are generalizable to most people with diagnosed dementia and sleep disturbance. Few patients were prescribed zaleplon or zolpidem and none eszopiclone; however, as they exert the same pharmacological action as zopiclone, the adverse effects of these agents are likely similar.

### Comparison with other studies

#### Fractures

Few studies have examined Z-drug dose and fracture risk. Greater risks of hip fracture were reported among older US care home residents taking higher dose Z-drugs, although limited by small numbers [45]. Additionally, few studies have examined fracture risk in PlwD taking Z-drugs. Consistent with our findings, a study of hospital records of PlwD in Japan reported increased fracture risks with Z-drug use, but they were unable to ascertain whether the prescription was given before or after the fracture [46]. Various studies report associations between Z-drugs and fracture risk, and specifically hip fracture risk, in older adults [12, 13]. However, our study and others suggest this relative risk is lower in PlwD [47, 48]. For example, in US nursing home residents, greater odds ratios were estimated between non-benzodiazepine hypnotic drug use and hip fracture in residents with no or only mild cognitive impairment than with moderate-severe cognitive impairment [47]. Z-drugs likely increase fracture risk through their effects on gait and balance [49, 50]. A randomised trial reported more tandem walk failures upon night-time awakening among older adults randomised to 5 mg zolpidem compared to placebo [51].

#### Falls

Z-drugs were originally claimed to cause fewer falls than benzodiazepines [52]; however, we found similar or larger effects. This is consistent with findings from older men in the Osteoporotic Fractures in Men study [53]. More fall-related injuries were observed with Z-drug use in older people in Taiwan, with greater frequencies when prescribed > 0.6 DDDs [54]. Increased fall rates have also been observed with dose increases in sedative-hypnotics in nursing home residents with dementia [55].

#### Cardiovascular outcomes

Similar to us, more strokes were observed in the Medicine use and Alzheimer’s disease (MEDALZ) cohort prescribed Z-drugs and adults in Taiwan prescribed zolpidem [15, 56]. Unfortunately, neither study estimated dose-specific risks. Mechanisms for Z-drugs causing increased stroke risk are uncertain, but could relate to decreased local cerebral blood flow [56]. However, as prolonged sleep disturbances likely increase stroke risk, residual confounding by sleep disturbance severity and duration could underlie reported associations [57].

#### Infections

Analysis of RCT data, generally in younger adults, indicated possible 1.5–2-fold increased infection risks when taking zopiclone and zolpidem [58]. We found inconsistent evidence of increased bacterial infection risk with higher dose Z-drugs. Other studies in older adults, including a MEDALZ cohort study, report no association between Z-drug use and risk of pneumonia [59–61]. Together, these suggest that if acute infection risk increases with higher dose Z-drug use in PlwD, then it is likely to be small, and our study was underpowered to detect it.

#### Mortality

Consistent with our findings, a MEDALZ cohort study found benzodiazepine use associated with increased mortality, but not Z-drugs [62]. Studies on Z-drug or benzodiazepine use and mortality in adults have been conflicting, and reported associations may simply stem from increased usage of benzodiazepines with approaching death [63].

#### Healthcare utilisation

We observed greater subsequent initiation of antipsychotics and antidepressants among Z-drug users, similarly observed by older people prescribed Z-drugs in a Taiwan study [54]. This likely reflects the increased behavioural and psychological symptoms of dementia as it progresses. The increased hospital visits we observed post-Z-drug initiation could partly reflect the increased fracture and stroke rates in these patients. The Taiwan study also reported greater rates of fall-related injuries requiring hospitalisation among Z-drug users [54].

## Conclusions

Higher doses of Z-drugs should be avoided in PlwD due to increased fracture and stroke risks. One in six PlwD in our study was commenced at 7.5 mg zopiclone or equivalent daily. Prescribers should use the lowest effective dose in the elderly and use simple specific drug regimens, and this advice needs implementing in national guidelines [64, 65]. Our findings suggest that the safety profile of Z-drugs should be considered similar to benzodiazepines in PlwD. Although the risks associated with low-dose Z-drugs were small, as the effectiveness of Z-drugs is also unproven in dementia, we advise adhering to the Beers criteria guidelines of avoiding Z-drug use in PlwD, where possible [66]. Alternative strategies should be sought for sleep disturbance other than Z-drug or benzodiazepine dose escalation. Where pharmacological management of sleep disturbance is initiated, fracture risk management plans are implemented, and prescriptions regularly reviewed to mitigate potential adverse health outcomes. This gives a clear and important steer for the use of hypnotics in people with dementia in clinical practice. This evidence is currently particularly important as social isolation related to the COVID-19 pandemic may increase the frequency of neuropsychiatric symptoms in dementia [67] and limit resources to offer non-pharmacological management approaches.

## Supplementary information

**Additional file 1.** Read codes to define dementia in CPRD, excluded patients and sleep disturbance.

**Additional file 2.** Dementia and sleep disturbance validation study.

**Additional file 3.** Read codes in CPRD and ICD-10 codes in HES and ONS to define the outcomes.

**Additional file 4.** Study protocol registration, Definition of covariates and Multiple Imputation Methods.

**Additional file 5.** Additional exclusion criteria, frequency of missing data, dose changes during follow-up, additional analysis results tables and flowchart of patient selection.

## Data Availability

This study is based on data from the Clinical Practice Research Datalink obtained under licence from the UK Medicines and Healthcare products Regulatory Agency (MHRA). However, the interpretation and conclusions contained in this report are those of the authors alone. Linked data from the Clinical Practice Research Datalink is available directly from CPRD. Full code lists for the covariates are available from the corresponding author at kathryn.richardson@uea.ac.uk.

## Abbreviations

ATC: Anatomical Therapeutic Chemical
BMI: Body mass index
CPRD: Clinical Practice Research Datalink
DDD: Defined daily doses
GP: General practitioner
HES: Hospital Episode Statistics
ICD-10: International classification of diseases 10th revision
MEDALZ: Medicine use and Alzheimer’s disease
NNH: Number needed to harm
ONS: Office of National Statistics
PlwD: People living with dementia
PPI: Patient and Public Involvement
TCA: Tricyclic antidepressant
