# Supplementary material for: Adverse effects of Z-drugs for sleep disturbance in people living with dementia: a population-based cohort study
Source: BMC Med. 2020 Nov 24;18:351. doi: 10.1186/s12916-020-01821-5 (PMC7683259; doi:10.1186/s12916-020-01821-5)
Supplement: Supplementary file 1 — Additional file 1. Read codes to define dementia in CPRD, excluded patients and sleep disturbance. [file 12916_2020_1821_MOESM1_ESM.docx]

**Additional file 1. Read codes to define dementia in CPRD, excluded patients and sleep disturbance**

*CPRD medcodes, corresponding Read codes and Read terms used to define dementia*

| medcode | Read code | Read Term |
| --- | --- | --- |
| 33707 | E00..00 | Senile and presenile organic psychotic conditions |
| 1916 | E00..11 | Senile dementia |
| 1350 | E00..12 | Senile/presenile dementia |
| 7323 | E000.00 | Uncomplicated senile dementia |
| 15165 | E001.00 | Presenile dementia |
| 42602 | E001000 | Uncomplicated presenile dementia |
| 49513 | E001100 | Presenile dementia with delirium |
| 30032 | E001200 | Presenile dementia with paranoia |
| 27677 | E001300 | Presenile dementia with depression |
| 38438 | E001z00 | Presenile dementia NOS |
| 44674 | E002.00 | Senile dementia with depressive or paranoid features |
| 18386 | E002000 | Senile dementia with paranoia |
| 21887 | E002100 | Senile dementia with depression |
| 41089 | E002z00 | Senile dementia with depressive or paranoid features NOS |
| 37015 | E003.00 | Senile dementia with delirium |
| 19477 | E004.00 | Arteriosclerotic dementia |
| 8634 | E004.11 | Multi infarct dementia |
| 43089 | E004000 | Uncomplicated arteriosclerotic dementia |
| 56912 | E004100 | Arteriosclerotic dementia with delirium |
| 55467 | E004200 | Arteriosclerotic dementia with paranoia |
| 43292 | E004300 | Arteriosclerotic dementia with depression |
| 42279 | E004z00 | Arteriosclerotic dementia NOS |
| 15249 | E00y.00 | Other senile and presenile organic psychoses |
| 51494 | E00y.11 | Presbyophrenic psychosis |
| 2882 | E00z.00 | Senile or presenile psychoses NOS |
| 62132 | E02y100 | Drug-induced dementia |
| 25386 | E041.00 | Dementia in conditions EC |
| 7664 | Eu00.00 | [X]Dementia in Alzheimer's disease |
| 49263 | Eu00000 | [X]Dementia in Alzheimer's disease with early onset |
| 25704 | Eu00011 | [X]Presenile dementia,Alzheimer's type |
| 60059 | Eu00012 | [X]Primary degen dementia, Alzheimer's type, presenile onset |
| 61528 | Eu00013 | [X]Alzheimer's disease type 2 |
| 38678 | Eu00100 | [X]Dementia in Alzheimer's disease with late onset |
| 46762 | Eu00111 | [X]Alzheimer's disease type 1 |
| 11379 | Eu00112 | [X]Senile dementia,Alzheimer's type |
| 43346 | Eu00113 | [X]Primary degen dementia of Alzheimer's type, senile onset |
| 30706 | Eu00200 | [X]Dementia in Alzheimer's dis, atypical or mixed type |
| 29386 | Eu00z00 | [X]Dementia in Alzheimer's disease, unspecified |
| 8195 | Eu00z11 | [X]Alzheimer's dementia unspec |
| 6578 | Eu01.00 | [X]Vascular dementia |
| 9565 | Eu01.11 | [X]Arteriosclerotic dementia |
| 46488 | Eu01000 | [X]Vascular dementia of acute onset |
| 11175 | Eu01100 | [X]Multi-infarct dementia |
| 55838 | Eu01111 | [X]Predominantly cortical dementia |
| 8934 | Eu01200 | [X]Subcortical vascular dementia |
| 31016 | Eu01300 | [X]Mixed cortical and subcortical vascular dementia |
| 55313 | Eu01y00 | [X]Other vascular dementia |
| 19393 | Eu01z00 | [X]Vascular dementia, unspecified |
| 12621 | Eu02.00 | [X]Dementia in other diseases classified elsewhere |
| 28402 | Eu02000 | [X]Dementia in Pick's disease |
| 54106 | Eu02100 | [X]Dementia in Creutzfeldt-Jakob disease |
| 37014 | Eu02200 | [X]Dementia in Huntington's disease |
| 9509 | Eu02300 | [X]Dementia in Parkinson's disease |
| 26270 | Eu02500 | [X]Lewy body dementia |
| 64267 | Eu02y00 | [X]Dementia in other specified diseases classif elsewhere |
| 4693 | Eu02z00 | [X] Unspecified dementia |
| 48501 | Eu02z11 | [X] Presenile dementia NOS |
| 47619 | Eu02z12 | [X] Presenile psychosis NOS |
| 34944 | Eu02z13 | [X] Primary degenerative dementia NOS |
| 4357 | Eu02z14 | [X] Senile dementia NOS |
| 27935 | Eu02z15 | [X] Senile psychosis NOS |
| 27759 | Eu02z16 | [X] Senile dementia, depressed or paranoid type |
| 53446 | Eu04100 | [X]Delirium superimposed on dementia |
| 1917 | F110.00 | Alzheimer's disease |
| 16797 | F110000 | Alzheimer's disease with early onset |
| 32057 | F110100 | Alzheimer's disease with late onset |
| 11136 | F111.00 | Pick's disease |
| 29512 | F112.00 | Senile degeneration of brain |
| 7572 | F116.00 | Lewy body disease |
| 59122 | Fyu3000 | [X]Other Alzheimer's disease |

*CPRD medcodes, corresponding Read codes and Read terms used to define patients excluded*

| medcode | Read code | Read term |
| --- | --- | --- |
| 1543 | PJ0..00 | Down's syndrome - trisomy 21 |
| 10759 | PJ0z.00 | Down's syndrome NOS |
| 18415 | PJ0..12 | Trisomy 21 |
| 23489 | PJ0..11 | Mongolism |
| 32010 | PJ01.00 | Trisomy 21, mosaicism |
| 42701 | PJ00.00 | Trisomy 21, meiotic nondisjunction |
| 61499 | PJ02.00 | Trisomy 21, translocation |
| 61627 | PJ0z.11 | Trisomy 21 NOS |
| 101309 | PJ02.11 | Partial trisomy 21 in Down's syndrome |
| 107919 | PJ01.11 | Trisomy 21, mitotic nondisjunction |
| 15958 | E1...00 | Non-organic psychoses |
| 854 | E10..00 | Schizophrenic disorders |
| 32222 | E100.00 | Simple schizophrenia |
| 73295 | E100.11 | Schizophrenia simplex |
| 15733 | E100000 | Unspecified schizophrenia |
| 3984 | E100200 | Chronic schizophrenic |
| 44498 | E100400 | Acute exacerbation of chronic schizophrenia |
| 58687 | E100500 | Schizophrenia in remission |
| 53625 | E100z00 | Simple schizophrenia NOS |
| 25546 | E102.00 | Catatonic schizophrenia |
| 102427 | E102500 | Catatonic schizophrenia in remission |
| 1494 | E103.00 | Paranoid schizophrenia |
| 33383 | E103000 | Unspecified paranoid schizophrenia |
| 31362 | E103200 | Chronic paranoid schizophrenia |
| 53032 | E103400 | Acute exacerbation of chronic paranoid schizophrenia |
| 36172 | E103500 | Paranoid schizophrenia in remission |
| 9281 | E103z00 | Paranoid schizophrenia NOS |
| 576 | E104.00 | Acute schizophrenic episode |
| 96883 | E105500 | Latent schizophrenia in remission |
| 38063 | E106.00 | Residual schizophrenia |
| 2117 | E107.00 | Schizo-affective schizophrenia |
| 58862 | E107000 | Unspecified schizo-affective schizophrenia |
| 43800 | E107200 | Chronic schizo-affective schizophrenia |
| 56438 | E107500 | Schizo-affective schizophrenia in remission |
| 10575 | E107z00 | Schizo-affective schizophrenia NOS |
| 33338 | E10y000 | Atypical schizophrenia |
| 49761 | E10yz00 | Other schizophrenia NOS |
| 8407 | E10z.00 | Schizophrenia NOS |
| 14656 | E11..00 | Affective psychoses |
| 8567 | E11..11 | Bipolar psychoses |
| 2560 | E11..12 | Depressive psychoses |
| 26161 | E11..13 | Manic psychoses |
| 37070 | E110.00 | Manic disorder, single episode |
| 18909 | E110.11 | Hypomanic psychoses |
| 20110 | E110000 | Single manic episode, unspecified |
| 14728 | E110100 | Single manic episode, mild |
| 70000 | E110600 | Single manic episode in full remission |
| 36611 | E110z00 | Manic disorder, single episode NOS |
| 26227 | E111.00 | Recurrent manic episodes |
| 19967 | E111000 | Recurrent manic episodes, unspecified |
| 32295 | E111400 | Recurrent manic episodes, severe, with psychosis |
| 37178 | E111600 | Recurrent manic episodes, in full remission |
| 46415 | E111z00 | Recurrent manic episode NOS |
| 32159 | E112400 | Single major depressive episode, severe, with psychosis |
| 24171 | E113400 | Recurrent major depressive episodes, severe, with psychosis |
| 3702 | E114.00 | Bipolar affective disorder, currently manic |
| 17385 | E114.11 | Manic-depressive - now manic |
| 35738 | E114000 | Bipolar affective disorder, currently manic, unspecified |
| 36126 | E114100 | Bipolar affective disorder, currently manic, mild |
| 46434 | E114200 | Bipolar affective disorder, currently manic, moderate |
| 63784 | E114600 | Bipolar affective disorder, currently manic, full remission |
| 4677 | E115.00 | Bipolar affective disorder, currently depressed |
| 12831 | E115.11 | Manic-depressive - now depressed |
| 35734 | E115100 | Bipolar affective disorder, currently depressed, mild |
| 27890 | E115200 | Bipolar affective disorder, currently depressed, moderate |
| 35607 | E115300 | Bipolar affect disord, now depressed, severe, no psychosis |
| 63701 | E115400 | Bipolar affect disord, now depressed, severe with psychosis |
| 57465 | E115600 | Bipolar affective disorder, now depressed, in full remission |
| 37296 | E115z00 | Bipolar affective disorder, currently depressed, NOS |
| 31316 | E116.00 | Mixed bipolar affective disorder |
| 31535 | E116000 | Mixed bipolar affective disorder, unspecified |
| 54195 | E116400 | Mixed bipolar affective disorder, severe, with psychosis |
| 63651 | E116500 | Mixed bipolar affective disorder, partial/unspec remission |
| 55064 | E116600 | Mixed bipolar affective disorder, in full remission |
| 63583 | E116z00 | Mixed bipolar affective disorder, NOS |
| 14784 | E117.00 | Unspecified bipolar affective disorder |
| 49763 | E117000 | Unspecified bipolar affective disorder, unspecified |
| 68647 | E117200 | Unspecified bipolar affective disorder, moderate |
| 24230 | E117600 | Unspecified bipolar affective disorder, in full remission |
| 27986 | E117z00 | Unspecified bipolar affective disorder, NOS |
| 60178 | E11y.00 | Other and unspecified manic-depressive psychoses |
| 11596 | E11y000 | Unspecified manic-depressive psychoses |
| 33426 | E11yz00 | Other and unspecified manic-depressive psychoses NOS |
| 41992 | E11z.00 | Other and unspecified affective psychoses |
| 54607 | E11z000 | Unspecified affective psychoses NOS |
| 33425 | E11zz00 | Other affective psychosis NOS |
| 4261 | E12..00 | Paranoid states |
| 14743 | E120.00 | Simple paranoid state |
| 3890 | E121.00 | Chronic paranoid psychosis |
| 14971 | E122.00 | Paraphrenia |
| 50868 | E123.11 | Folie a deux |
| 31589 | E12y.00 | Other paranoid states |
| 31455 | E12yz00 | Other paranoid states NOS |
| 12771 | E12z.00 | Paranoid psychosis NOS |
| 31984 | E13..00 | Other nonorganic psychoses |
| 20228 | E13..11 | Reactive psychoses |
| 8478 | E130.00 | Reactive depressive psychosis |
| 17770 | E130.11 | Psychotic reactive depression |
| 29937 | E131.00 | Acute hysterical psychosis |
| 7332 | E132.00 | Reactive confusion |
| 15053 | E133.00 | Acute paranoid reaction |
| 24345 | E134.00 | Psychogenic paranoid psychosis |
| 16333 | E13y.00 | Other reactive psychoses |
| 14965 | E13z.00 | Nonorganic psychosis NOS |
| 3636 | E13z.11 | Psychotic episode NOS |
| 22188 | E1z..00 | Non-organic psychosis NOS |
| 61969 | E212200 | Schizotypal personality |
| 17281 | Eu2..00 | [X]Schizophrenia, schizotypal and delusional disorders |
| 34236 | Eu20.00 | [X]Schizophrenia |
| 16764 | Eu20000 | [X]Paranoid schizophrenia |
| 35877 | Eu20213 | [X]Schizophrenic catatonia |
| 20785 | Eu20400 | [X]Post-schizophrenic depression |
| 24107 | Eu20511 | [X]Chronic undifferentiated schizophrenia |
| 35848 | Eu20600 | [X]Simple schizophrenia |
| 49420 | Eu20y00 | [X]Other schizophrenia |
| 94001 | Eu20y12 | [X]Schizophreniform disord NOS |
| 18053 | Eu20y13 | [X]Schizophrenifrm psychos NOS |
| 34966 | Eu20z00 | [X]Schizophrenia, unspecified |
| 39316 | Eu21.00 | [X]Schizotypal disorder |
| 26859 | Eu21.18 | [X]Schizotypal personality disorder |
| 28562 | Eu22.00 | [X]Persistent delusional disorders |
| 34389 | Eu22000 | [X]Delusional disorder |
| 2113 | Eu22011 | [X]Paranoid psychosis |
| 11172 | Eu22012 | [X]Paranoid state |
| 47947 | Eu22013 | [X]Paraphrenia – late |
| 4843 | Eu22015 | [X]Paranoia |
| 62405 | Eu22100 | [X]Delusional misidentification syndrome |
| 55221 | Eu22111 | [X]Capgras syndrome |
| 101720 | Eu22300 | [X]Paranoid state in remission |
| 40981 | Eu22y11 | [X]Delusional dysmorphophobia |
| 50248 | Eu22y12 | [X]Involutional paranoid state |
| 49223 | Eu22z00 | [X]Persistent delusional disorder, unspecified |
| 25019 | Eu23.00 | [X]Acute and transient psychotic disorders |
| 36720 | Eu23000 | [X]Acute polymorphic psychot disord without symp of schizoph |
| 21455 | Eu23012 | [X]Cycloid psychosis |
| 26143 | Eu23112 | [X]Cycloid psychosis with symptoms of schizophrenia |
| 44307 | Eu23300 | [X]Other acute predominantly delusional psychotic disorders |
| 27770 | Eu23312 | [X]Psychogenic paranoid psychosis |
| 44503 | Eu23y00 | [X]Other acute and transient psychotic disorders |
| 34168 | Eu23z00 | [X]Acute and transient psychotic disorder, unspecified |
| 31707 | Eu23z11 | [X]Brief reactive psychosis NOS |
| 29651 | Eu23z12 | [X]Reactive psychosis |
| 105606 | Eu24.11 | [X]Folie a deux |
| 11973 | Eu24.13 | [X]Induced psychotic disorder |
| 9422 | Eu25.00 | [X]Schizoaffective disorders |
| 33847 | Eu25000 | [X]Schizoaffective disorder, manic type |
| 16905 | Eu25011 | [X]Schizoaffective psychosis, manic type |
| 51903 | Eu25012 | [X]Schizophreniform psychosis, manic type |
| 11055 | Eu25100 | [X]Schizoaffective disorder, depressive type |
| 35274 | Eu25111 | [X]Schizoaffective psychosis, depressive type |
| 33693 | Eu25200 | [X]Schizoaffective disorder, mixed type |
| 37580 | Eu25212 | [X]Mixed schizophrenic and affective psychosis |
| 58532 | Eu25y00 | [X]Other schizoaffective disorders |
| 37681 | Eu25z00 | [X]Schizoaffective disorder, unspecified |
| 33410 | Eu25z11 | [X]Schizoaffective psychosis NOS |
| 101987 | Eu26.00 | [X]Nonorganic psychosis in remission |
| 30985 | Eu2y.00 | [X]Other nonorganic psychotic disorders |
| 31738 | Eu2y.11 | [X]Chronic hallucinatory psychosis |
| 11244 | Eu2z.00 | [X]Unspecified nonorganic psychosis |
| 694 | Eu2z.11 | [X]Psychosis NOS |
| 5726 | Eu3..00 | [X]Mood - affective disorders |
| 12173 | Eu30.00 | [X]Manic episode |
| 9521 | Eu30.11 | [X]Bipolar disorder, single manic episode |
| 2741 | Eu30000 | [X]Hypomania |
| 13024 | Eu30100 | [X]Mania without psychotic symptoms |
| 21065 | Eu30200 | [X]Mania with psychotic symptoms |
| 48632 | Eu30212 | [X]Mania with mood-incongruent psychotic symptoms |
| 32088 | Eu30y00 | [X]Other manic episodes |
| 44513 | Eu30z00 | [X]Manic episode, unspecified |
| 4678 | Eu30z11 | [X]Mania NOS |
| 6874 | Eu31.00 | [X]Bipolar affective disorder |
| 1531 | Eu31.11 | [X]Manic-depressive illness |
| 6710 | Eu31.12 | [X]Manic-depressive psychosis |
| 16808 | Eu31000 | [X]Bipolar affective disorder, current episode hypomanic |
| 26299 | Eu31100 | [X]Bipolar affect disorder cur epi manic wout psychotic symp |
| 28277 | Eu31200 | [X]Bipolar affect disorder cur epi manic with psychotic symp |
| 16562 | Eu31300 | [X]Bipolar affect disorder cur epi mild or moderate depressn |
| 23713 | Eu31400 | [X]Bipol aff disord, curr epis sev depress, no psychot symp |
| 4732 | Eu31500 | [X]Bipolar affect dis cur epi severe depres with psyc symp |
| 27584 | Eu31700 | [X]Bipolar affective disorder, currently in remission |
| 103915 | Eu31900 | [X]Bipolar affective disorder type II |
| 53840 | Eu31y00 | [X]Other bipolar affective disorders |
| 73924 | Eu31y11 | [X]Bipolar II disorder |
| 51032 | Eu31y12 | [X]Recurrent manic episodes |
| 33751 | Eu31z00 | [X]Bipolar affective disorder, unspecified |
| 12099 | Eu32300 | [X]Severe depressive episode with psychotic symptoms |
| 24117 | Eu32311 | [X]Single episode of major depression and psychotic symptoms |
| 52678 | Eu32312 | [X]Single episode of psychogenic depressive psychosis |
| 24112 | Eu32313 | [X]Single episode of psychotic depression |
| 28863 | Eu32314 | [X]Single episode of reactive depressive psychosis |
| 98417 | Eu32800 | [X]Major depression, severe with psychotic symptoms |
| 29451 | Eu33213 | [X]Manic-depress psychosis,depressd,no psychotic symptoms |
| 47009 | Eu33300 | [X]Recurrent depress disorder cur epi severe with psyc symp |
| 23731 | Eu33311 | [X]Endogenous depression with psychotic symptoms |
| 28677 | Eu33312 | [X]Manic-depress psychosis,depressed type+psychotic symptoms |
| 32941 | Eu33313 | [X]Recurr severe episodes/major depression+psychotic symptom |
| 31757 | Eu33314 | [X]Recurr severe episodes/psychogenic depressive psychosis |
| 16861 | Eu33315 | [X]Recurrent severe episodes of psychotic depression |
| 37764 | Eu33316 | [X]Recurrent severe episodes/reactive depressive psychosis |
| 31633 | Eu3z.11 | [X]Affective psychosis NOS |
| 28168 | Eu44.14 | [X]Hysterical psychosis |
| 8430 | 1462 | H/O: alcoholism |
| 16237 | E01..00 | Alcoholic psychoses |
| 16225 | E010.00 | Alcohol withdrawal delirium |
| 22277 | E010.11 | DTs - delirium tremens |
| 1476 | E010.12 | Delirium tremens |
| 20762 | E011.00 | Alcohol amnestic syndrome |
| 4500 | E011000 | Korsakov's alcoholic psychosis |
| 11106 | E011100 | Korsakov's alcoholic psychosis with peripheral neuritis |
| 18636 | E011200 | Wernicke-Korsakov syndrome |
| 41920 | E011z00 | Alcohol amnestic syndrome NOS |
| 54505 | E012.00 | Other alcoholic dementia |
| 27342 | E012.11 | Alcoholic dementia NOS |
| 37946 | E012000 | Chronic alcoholic brain syndrome |
| 25110 | E013.00 | Alcohol withdrawal hallucinosis |
| 57939 | E014.00 | Pathological alcohol intoxication |
| 20407 | E014.11 | Drunkenness – pathological |
| 30404 | E015.00 | Alcoholic paranoia |
| 33670 | E01y.00 | Other alcoholic psychosis |
| 2082 | E01y000 | Alcohol withdrawal syndrome |
| 68111 | E01yz00 | Other alcoholic psychosis NOS |
| 67651 | E01z.00 | Alcoholic psychosis NOS |
| 2084 | E23..00 | Alcohol dependence syndrome |
| 2081 | E23..11 | Alcoholism |
| 1399 | E23..12 | Alcohol problem drinking |
| 5740 | E230.00 | Acute alcoholic intoxication in alcoholism |
| 57714 | E230.11 | Alcohol dependence with acute alcoholic intoxication |
| 40530 | E230000 | Acute alcoholic intoxication, unspecified, in alcoholism |
| 56947 | E230100 | Continuous acute alcoholic intoxication in alcoholism |
| 21624 | E230200 | Episodic acute alcoholic intoxication in alcoholism |
| 59574 | E230300 | Acute alcoholic intoxication in remission, in alcoholism |
| 36296 | E230z00 | Acute alcoholic intoxication in alcoholism NOS |
| 31443 | E231.00 | Chronic alcoholism |
| 37605 | E231.11 | Dipsomania |
| 43193 | E231000 | Unspecified chronic alcoholism |
| 24064 | E231100 | Continuous chronic alcoholism |
| 26106 | E231200 | Episodic chronic alcoholism |
| 24485 | E231300 | Chronic alcoholism in remission |
| 33635 | E231z00 | Chronic alcoholism NOS |
| 6169 | E23z.00 | Alcohol dependence syndrome NOS |
| 39327 | Eu10200 | [X]Mental and behav dis due to use alcohol: dependence syndr |
| 28780 | Eu10211 | [X]Alcohol addiction |
| 5758 | Eu10212 | [X]Chronic alcoholism |
| 69691 | Eu10213 | [X]Dipsomania |
| 20514 | Eu10300 | [X]Mental and behav dis due to use alcohol: withdrawal state |
| 64101 | Eu10400 | [X]Men & behav dis due alcohl: withdrawl state with delirium |
| 17259 | Eu10411 | [X]Delirium tremens, alcohol induced |
| 6467 | Eu10511 | [X]Alcoholic hallucinosis |
| 65932 | Eu10512 | [X]Alcoholic jealousy |
| 30162 | Eu10513 | [X]Alcoholic paranoia |
| 17607 | Eu10514 | [X]Alcoholic psychosis NOS |
| 11670 | Eu10611 | [X]Korsakov's psychosis, alcohol induced |
| 26323 | Eu10711 | [X]Alcoholic dementia NOS |
| 37691 | Eu10712 | [X]Chronic alcoholic brain syndrome |
| 47555 | F11x000 | Cerebral degeneration due to alcoholism |
| 36748 | F11x011 | Alcoholic encephalopathy |
| 33839 | F144000 | Cerebellar ataxia due to alcoholism |
| 2925 | F375.00 | Alcoholic polyneuropathy |
| 31742 | F394100 | Alcoholic myopathy |
| 7603 | Fy03.00 | Sleep apnoea |
| 8148 | Fy03.11 | Obstructive sleep apnoea |
| 38686 | Fy04.00 | Sleep-related respiratory failure |
| 59155 | Fy04.11 | Ondine's curse |
| 4915 | G555.00 | Alcoholic cardiomyopathy |
| 8363 | G852300 | Oesophageal varices in alcoholic cirrhosis of the liver |
| 23779 | H5B..00 | Sleep apnoea |
| 20748 | H5B0.00 | Obstructive sleep apnoea |
| 4506 | J153.00 | Alcoholic gastritis |
| 10691 | J610.00 | Alcoholic fatty liver |
| 3216 | J611.00 | Acute alcoholic hepatitis |
| 4743 | J612.00 | Alcoholic cirrhosis of liver |
| 21713 | J612000 | Alcoholic fibrosis and sclerosis of liver |
| 7885 | J613.00 | Alcoholic liver damage unspecified |
| 17330 | J613000 | Alcoholic hepatic failure |
| 7943 | J617.00 | Alcoholic hepatitis |
| 7602 | J617000 | Chronic alcoholic hepatitis |
| 24984 | J671000 | Alcohol-induced chronic pancreatitis |
| 48539 | R005100 | [D]Insomnia with sleep apnoea |
| 36301 | R005300 | [D]Hypersomnia with sleep apnoea |
| 2506 | R005311 | [D]Sleep apnoea syndrome |
| 20438 | R005312 | [D]Syndrome sleep apnoea |
| 7123 | ZV11300 | [V]Personal history of |
| 1598 | A531.11 | Post-herpetic neuralgia |
| 27403 | A531100 | Geniculate herpes zoster |
| 7331 | A531111 | Ramsay - Hunt syndrome |
| 11498 | A531200 | Postherpetic trigeminal neuralgia |
| 31709 | A531300 | Postherpetic polyneuropathy |
| 17180 | A531500 | Postzoster neuralgia |
| 10223 | A531511 | Postherpetic neuralgia |
| 28333 | C373200 | Familial neuropathic amyloid |
| 7584 | F300.00 | Post-herpetic trigeminal neuralgia |
| 1541 | F301.00 | Other specified trigeminal neuralgia |
| 4912 | F301000 | Tic douloureux |
| 6581 | F301z00 | Trigeminal neuralgia NOS |
| 18016 | F336000 | Phantom limb syndrome with pain |
| 23768 | F337.00 | Nerve root and plexus compressions in diseases EC |
| 55335 | F337000 | Nerve root and plexus compressions in neoplastic disease |
| 33604 | F337100 | Nerve root and plexus compressions in intervert disc disord |
| 23699 | F337200 | Nerve root and plexus compressions in spondylosis |
| 24410 | F337300 | Nerve root and plexus compressions in other dorsopathies |
| 56272 | F374.00 | Polyneuropathy in disease EC |
| 39692 | F374400 | Polyneuropathy in herpes zoster |
| 63555 | F374z00 | Polyneuropathy in disease NOS |
| 93868 | Fyu6A00 | [X]Other mononeuropathies of upper limb |
| 72922 | Fyu6B00 | [X]Other mononeuropathies of lower limb |
| 91741 | Fyu6C00 | [X]Other specified mononeuropathies |
| 107322 | Fyu6D00 | [X]Other mononeuropathies in diseases classified elsewhere |
| 22238 | Fyu6E00 | [X]Ilio-inguinal nerve entrapment |
| 55076 | Fyu7.00 | [X]Polyneuropathies & other disord of peripheral nerv syst |
| 97449 | Fyu7000 | [X]Other hereditary and idiopathic neuropathies |
| 97479 | Fyu7100 | [X]Other inflammatory polyneuropathies |
| 97306 | Fyu7200 | [X]Other specified polyneuropathies |
| 39858 | Fyu7B00 | [X]Inflammatory polyneuropathy, unspecified |
| 35537 | Fyu7C00 | [X] Polyneuropathy, unspecified |
| 99855 | M271700 | Neuropathic foot ulcer |
| 49575 | N035.00 | Neuropathic arthropathy |
| 8710 | N035.11 | Charcot's arthropathy |
| 36643 | N035.12 | Neuropathic arthritis |
| 37759 | N11y200 | Neuropathic spondylopathy \| |
| 54992 | N242.00 | Neuralgia, neuritis and radiculitis unspecified |
| 2284 | N242000 | Neuralgia unspecified |
| 1416 | N242100 | Neuritis unspecified |
| 769 | N242200 | Radiculitis unspecified |
| 11544 | N242300 | Neuropathic pain |
| 23839 | N242z00 | Neuralgia, neuritis or radiculitis NOS |
| 41736 | N242z11 | Policeman's disease |
| 18492 | SD72200 | Neuropathic foot blister |

*CPRD medcodes, corresponding Read codes and Read terms used to define sleep disturbance*

| Medcode | Read code | Read term |
| --- | --- | --- |
| 21305 | 1B1B.00 | Cannot sleep - insomnia |
| 4537 | 1B1B.11 | C/O - insomnia |
| 3523 | 1B1B000 | Initial insomnia |
| 5675 | 1B1B100 | Middle insomnia |
| 4597 | 1B1B200 | Late insomnia |
| 7725 | 1B1Q.00 | Poor sleep pattern |
| 30385 | 1BN1.00 | Wanders at night |
| 94508 | 1BN2.00 | Wanders during the day and at night |
| 42847 | 1BX0.00 | Delayed onset of sleep |
| 60806 | 1BX2.00 | Sleeping pattern |
| 60974 | 1BX9.00 | Light sleep |
| 96037 | 8G9B.00 | Sleep hygiene behaviour education |
| 95887 | 8HTn.00 | Referral to sleep clinic |
| 12072 | 8Q0..00 | Sleep management |
| 107666 | 9Ngt.00 | On melatonin for sleep disorder |
| 7819 | E274.00 | Non-organic sleep disorders |
| 26546 | E274.12 | Insomnia due to nonorganic sleep disorder |
| 15515 | E274100 | Transient insomnia |
| 4023 | E274111 | Insomnia NOS |
| 16115 | E274200 | Persistent insomnia |
| 39990 | E274B00 | Repeated rapid eye movement sleep interruptions |
| 55179 | E274C00 | Other sleep stage or arousal dysfunction |
| 19514 | E274D11 | Restless sleep |
| 32987 | E274E00 | 'Short-sleeper' |
| 36745 | E274F00 | Inversion of sleep rhythm |
| 30626 | Eu51000 | [X]Nonorganic insomnia |
| 23923 | Eu51200 | [X]Nonorganic disorder of the sleep-wake schedule |
| 42753 | Eu51211 | [X]Psychogenic inversion of circadian rhythm |
| 101729 | Eu51213 | [X]Psychogenic inversion of sleep rhythm |
| 5921 | Fy00.00 | Disorders of initiating and maintaining sleep |
| 8997 | Fy02.00 | Disorders of the sleep-wake schedule |
| 8084 | R005.00 | [D]Sleep disturbances |
| 10349 | R005.11 | [D]Insomnia - symptom |
| 31236 | R005.12 | [D]Sleep rhythm problems |
| 750 | R005200 | [D]Insomnia NOS |
| 16447 | R005500 | [D]Sleep rhythm inversion |
| 15732 | R005600 | [D]Sleep rhythm irregular |
| 58911 | R005700 | [D]Sleep-wake rhythm non-24-hour cycle |
| 54458 | R005800 | [D]Sleep dysfunction with sleep stage disturbance |
| 22081 | Z1M..00 | Sleep and rest interventions |
| 101913 | Z1M1.00 | Disturbing sleep |
| 51397 | ZV1B100 | [V]Personal history of unhealthy sleep-wake schedule |
| 43397 | Z7CCC00 | Found wandering the streets |
| 26009 | Z7CCB00 | Wandering |
| 15407 | R005z00 | [D]Sleep dysfunction NOS |
| 41737 | R005900 | [D]Sleep dysfunction with arousal disturbance |
| 1244 | R005000 | [D]Sleep disturbance, unspecified |
| 15283 | K5A2100 | Menopausal sleeplessness |
| 53912 | Fyu5800 | [X]Other sleep disorders |
| 49601 | Fy05.00 | Nocturnal sleep-related eating disorder |
| 2329 | Fy0..00 | Sleep disorders |
| 21032 | Eu51z11 | [X]Emotional sleep disorder NOS |
| 22819 | Eu51z00 | [X]Nonorganic sleep disorder, unspecified |
| 62925 | Eu51y00 | [X]Other nonorganic sleep disorders |
| 17687 | Eu51511 | [X]Dream anxiety disorder |
| 6943 | Eu51300 | [X]Sleepwalking |
| 24894 | Eu51.00 | [X]Nonorganic sleep disorders |
| 27649 | E274z00 | Non-organic sleep disorder NOS |
| 8519 | E274y11 | Dreams |
| 43098 | E274y00 | Other non-organic sleep disorder |
| 48783 | E274D00 | Repetitive intrusions of sleep |
| 47745 | E274600 | Shifting sleep-work schedule |
| 7409 | E274500 | Jet lag syndrome |
| 36992 | E274300 | Transient hypersomnia |
| 16434 | E274000 | Unspecified non-organic sleep disorder |
| 93615 | 9Nk0.00 | Seen in sleep clinic |
| 103449 | 1F9C.00 | Eats at night |
| 9090 | 1BN..00 | Wandering |
| 8123 | 1B1O.00 | Restless |
| 56809 | 7065800 | Sleep studies |
| 4559 | 3148 | Sleep studies |
