# Supplementary material for: Adverse effects of Z-drugs for sleep disturbance in people living with dementia: a population-based cohort study
Source: BMC Med. 2020 Nov 24;18:351. doi: 10.1186/s12916-020-01821-5 (PMC7683259; doi:10.1186/s12916-020-01821-5)
Supplement: Supplementary file 2 — Additional file 2. Dementia and sleep disturbance validation study. [file 12916_2020_1821_MOESM2_ESM.docx]

**Additional file 2. Dementia and sleep disturbance validation study**

To validate the accuracy of the coding for our patient selection, we sent a questionnaire to the GP practices of 106 patients, asking whether the patient had a dementia diagnosis or sleep disturbance record (yes or no). The randomly selected patients were registered with their GP in 2017 and either prescribed Z-drugs, low-dose TCAs, or recorded as having sleep disturbance but not prescribed sedative-hypnotics. We subsequently, no longer examined patients exposed to low-dose TCAs (see additional file 4 for protocol registration and deviations).

**Results**

A total of 56 (53%) GPs completed our validation questionnaire. GP practices confirmed the dementia diagnosis for 54 (96%) patients. Sleep disturbance was confirmed for 18 out of 22 (82%) and eight out of 19 (42%) patients in the Z-drug and sleep disturbance (no sedative-hypnotic) cohorts. Our further inspection of the CPRD records revealed that when specific Read codes for “Sleep disturbances”, “Insomnia Not Otherwise Specified” or “Sleep management” were entered, a further pop-up screen in the software used by practices ask the GP to record the patient’s “sleep pattern” (with the first option being “satisfactory”) and the average hours sleep per night. Hence giving uncertainty as to whether sometimes the GPs were using the Read codes “Sleep disturbances”, “Insomnia Not Otherwise Specified” or “Sleep management” to record a good night’s sleep. GP practices confirmed sleep disturbance among three (60%) of the five sleep disturbance patients where the GP did not record a “satisfactory” sleep pattern. GP practices confirmed sleep disturbance among eight (53%) of the 15 sleep disturbance patients where the GP did not record greater than six hours of sleep per night.
