# Supplementary material for: Adverse effects of Z-drugs for sleep disturbance in people living with dementia: a population-based cohort study
Source: BMC Med. 2020 Nov 24;18:351. doi: 10.1186/s12916-020-01821-5 (PMC7683259; doi:10.1186/s12916-020-01821-5)
Supplement: Supplementary file 3 — Additional file 3. Read codes in CPRD and ICD-10 codes in HES and ONS to define the outcomes. [file 12916_2020_1821_MOESM3_ESM.docx]

**Additional file 3. Read codes in CPRD and ICD-10 codes in HES and ONS to define the outcomes**

Read codes used to identify hip fractures

| **medcode** | **Read code** | **Read term** |
| --- | --- | --- |
| 9792 | 7K1D01E | DHS - Dynamic hip screw primary fixation of neck of femur |
| 12544 | 7K1D01F | Dynamic hip screw primary fixation of neck of femur |
| 6660 | 7K1L400 | Closed reduction of fracture of hip |
| 2225 | S30..00 | Fracture of neck of femur |
| 1994 | S30..11 | Hip fracture |
| 19387 | S302011 | Closed fracture of femur, greater trochanter |
| 8648 | S302400 | Closed fracture of femur, intertrochanteric |
| 8243 | S305.00 | Subtrochanteric fracture |
| 24276 | S30w.00 | Closed fracture of unspecified proximal femur |
| 18273 | S30y.00 | Closed fracture of neck of femur NOS |
| 10570 | S30y.11 | Hip fracture NOS |

Read codes used to identify forearm fracture

| **medcode** | **Read code** | **Read term** |
| --- | --- | --- |
| 8885 | 7K1LJ00 | Closed reduction of fracture of thumb |
| 6942 | 7K1LL00 | Closed reduction of fracture of radius and or ulna |
| 5951 | 7K1LM00 | Closed reduction of fracture of wrist |
| 1250 | S224.11 | Elbow fracture - closed |
| 6825 | S23..00 | Fracture of radius and ulna |
| 43570 | S230.00 | Closed fracture of proximal radius and ulna |
| 7009 | S230600 | Closed fracture radius, head |
| 18299 | S234.00 | Closed fracture of radius and ulna, lower end |
| 203 | S234.11 | Wrist fracture - closed |
| 343 | S234100 | Closed Colles' fracture |
| 1742 | S234200 | Closed fracture of the distal radius, unspecified |
| 9165 | S234300 | Closed fracture of ulna, styloid process |
| 40476 | S234500 | Closed fracture distal ulna, unspecified |
| 199 | S23B.00 | Fracture of lower end of radius |
| 137 | S23x111 | Fracture of radius NOS |
| 1073 | S23x211 | Fracture of ulna NOS |
| 909 | S23z.00 | Fracture of radius and ulna, NOS |
| 22375 | S24..00 | Fracture of carpal bone |
| 15666 | S240.00 | Closed fracture of carpal bone |
| 8056 | S242.00 | Fracture at wrist and hand level |
| 553 | S242000 | Fracture of scaphoid |
| 993 | S242200 | Fracture of other metacarpal bone |
| 25519 | S250300 | Closed fracture finger metacarpal shaft |
| 12546 | S250400 | Closed fracture finger metacarpal neck |
| 29111 | S251.00 | Open fracture of metacarpal bone(s) |
| 441 | S26..00 | Fracture of one or more phalanges of hand |
| 5260 | S26..11 | Finger fracture |
| 8302 | S260.00 | Closed fracture of one or more phalanges of hand |
| 24516 | S260D00 | Closed fracture finger proximal phalanx |
| 7500 | S262.00 | Fracture of thumb |
| 6299 | S263.00 | Fracture of other finger |
| 4582 | S26z.00 | Fracture of one or more phalanges of hand NOS |
| 18614 | S4C..00 | Fracture-dislocation or subluxation of wrist |
| 10250 | S4D..00 | Fracture-dislocation/subluxation finger/thumb |

Fracture- Any of the above codes for hip or forearm fracture, plus any of the following codes

| **medcode** | **Read code** | **Read term** |
| --- | --- | --- |
| 18962 | 7K1L500 | Closed reduction of fracture of femur |
| 6106 | 7K1L800 | Closed reduction of fracture of ankle |
| 7339 | 7K1LA00 | Closed reduction of fracture of toe |
| 6379 | 7K1LF00 | Closed reduction of fracture of humerus |
| 7428 | 7K1LG00 | Closed reduction of fracture of shoulder |
| 5526 | N331.00 | Pathological fracture |
| 30616 | N331000 | Pathological fracture of thoracic vertebra |
| 17377 | N331800 | Osteoporosis + pathological fracture lumbar vertebrae |
| 12673 | N331900 | Osteoporosis + pathological fracture thoracic vertebrae |
| 11543 | N331G00 | Collapse of lumbar vertebra |
| 45736 | N331H00 | Collapse of cervical vertebra due to osteoporosis |
| 4013 | N331L00 | Collapse of vertebra due to osteoporosis NOS |
| 11503 | N331M00 | Fragility fracture due to unspecified osteoporosis |
| 93497 | N331N00 | Fragility fracture |
| 4409 | S10..12 | Fracture of vertebra without spinal cord lesion |
| 11296 | S100.00 | Closed fracture of cervical spine |
| 39887 | S100A00 | Closed fracture axis, odontoid process |
| 3888 | S104.00 | Closed fracture lumbar vertebra |
| 8266 | S104100 | Closed fracture lumbar vertebra, wedge |
| 34403 | S10A100 | Fracture of second cervical vertebra |
| 9072 | S10B400 | Fracture of acetabulum |
| 280 | S120.00 | Closed fracture rib |
| 7831 | S120000 | Closed fracture of rib, unspecified |
| 9688 | S127.00 | Fracture of rib |
| 738 | S13..00 | Fracture or disruption of pelvis |
| 5302 | S132.00 | Closed fracture pubis |
| 7004 | S132000 | Closed fracture pelvis, single pubic ramus |
| 6667 | S132100 | Closed fracture pelvis, multiple pubic rami - stable |
| 46592 | S132200 | Closed fracture pelvis, multiple pubic rami - unstable |
| 28702 | S132z00 | Closed fracture pubis NOS |
| 28375 | S13y.00 | Closed fracture of pelvis NOS |
| 11277 | S150.00 | Multiple fractures of thoracic spine |
| 6195 | S2...00 | Fracture of upper limb |
| 5929 | S2...11 | Arm fracture |
| 483 | S20..00 | Fracture of clavicle |
| 44715 | S200000 | Closed fracture of clavicle, unspecified part |
| 29899 | S200300 | Closed fracture clavicle, lateral end |
| 1177 | S21..00 | Fracture of scapula |
| 10735 | S21..11 | Shoulder blade fracture |
| 517 | S22..00 | Fracture of humerus |
| 11222 | S220.00 | Closed fracture of the proximal humerus |
| 44721 | S220000 | Closed fracture of proximal humerus, unspecified part |
| 11313 | S220100 | Closed fracture proximal humerus, neck |
| 11044 | S220300 | Closed fracture proximal humerus, greater tuberosity |
| 6893 | S224100 | Closed fracture distal humerus, supracondylar |
| 1548 | S228.00 | Fracture of lower end of humerus |
| 10382 | S22z.00 | Fracture of humerus NOS |
| 8891 | S3...00 | Fracture of lower limb |
| 8040 | S31..00 | Other fracture of femur |
| 6868 | S310.00 | Closed fracture of femur, shaft or unspecified part |
| 37662 | S310000 | Closed fracture of femur, unspecified part |
| 12791 | S310011 | Thigh fracture NOS |
| 6320 | S312100 | Closed fracture of femoral condyle, unspecified |
| 5332 | S312300 | Closed fracture distal femur, supracondylar |
| 8646 | S314.00 | Fracture of shaft of femur |
| 8589 | S315.00 | Fracture of lower end of femur |
| 520 | S31z.00 | Fracture of femur, NOS |
| 235 | S32..00 | Fracture of patella |
| 27719 | S334.00 | Closed fracture distal tibia |
| 6839 | S339000 | Closed fracture of distal fibula |
| 78444 | S33A.00 | Fracture of tibia |
| 4304 | S33x100 | Closed fracture of fibula, unspecified part, NOS |
| 4572 | S33x200 | Closed fracture of tibia and fibula, unspecified part |
| 325 | S34..00 | Fracture of ankle |
| 7135 | S342000 | Closed fracture ankle, lateral malleolus, low |
| 7317 | S344.00 | Closed fracture ankle, bimalleolar |
| 169 | S35..11 | Metatarsal bone fracture |
| 2710 | S35..12 | Tarsal bone fracture |
| 8276 | S350.00 | Closed fracture of calcaneus |
| 3937 | S352700 | Closed fracture metatarsal |
| 35077 | S352E00 | Closed fracture metatarsal head |
| 6062 | S356.00 | Fracture of metatarsal bone |
| 2176 | S362.00 | Fracture of great toe |
| 2470 | S3z..00 | Fracture of unspecified bones |
| 358 | S3z..11 | Fracture NOS |
| 34212 | S4J2100 | Closed fracture-subluxation of pelvis |

Read codes used to identify falls

| **medcode** | **Read code** | **Read term** |
| --- | --- | --- |
| 6008 | 16D..00 | Falls |
| 8694 | 16D1.00 | Recurrent falls |
| 46559 | 16D2.00 | Number of falls in last year |
| 98223 | 16D5.00 | Fall onto outstretched hand |
| 108062 | 16D6.00 | Fall |
| 5284 | 1B65.00 | Had a collapse |
| 1634 | 1B65.11 | Collapse - symptom |
| 10412 | 224..00 | O/E - collapsed |
| 105499 | 8CMW400 | Falls care pathway |
| 2307 | R002.00 | [D]Syncope and collapse |
| 1812 | R002300 | [D]Collapse |
| 4859 | R200.12 | [D] Geriatric fall |
| 6815 | TC...00 | Accidental falls |
| 384 | TC...11 | Fall - accidental |
| 38818 | TC42000 | Fall from chair |
| 26432 | TC42100 | Fall from bed |
| 15112 | TC5..00 | Fall on same level from slipping, tripping or stumbling |
| 18007 | TC50.00 | Fall on same level from slipping |
| 7948 | TC52.00 | Fall on same level from stumbling |
| 8730 | TCy..00 | Other falls |
| 11308 | TCyz.00 | Other accidental fall NOS |
| 6835 | TCz..00 | Accidental falls NOS |
| 7970 | U10..00 | [X]Falls |
| 43191 | U10J000 | [X]Other fall on same level, occurrence at home |
| 24776 | U10z.00 | [X]Unspecified fall |

Read codes used to identify acute bacterial infection

| **medcode** | | **Read code** | | **Read term** | |
| --- | --- | --- | --- | --- | --- |
| 23640 | | H0y..00 | | Other specified acute respiratory infections | |
| 21113 | | H0z..00 | | Acute respiratory infection NOS | |
| 10086 | | H2...00 | | Pneumonia and influenza | |
| 1849 | | H21..00 | | Lobar (pneumococcal) pneumonia | |
| 12061 | | H22y200 | | Pneumonia - Legionella | |
| 23095 | | H22z.00 | | Bacterial pneumonia NOS | |
| 25694 | | H23..00 | | Pneumonia due to other specified organisms | |
| 886 | | H25..00 | | Bronchopneumonia due to unspecified organism | |
| 16287 | | H25..11 | | Chest infection - unspecified bronchopneumonia | |
| 572 | | H26..00 | | Pneumonia due to unspecified organism | |
| 9639 | | H260.00 | | Lobar pneumonia due to unspecified organism | |
| 3683 | | H261.00 | | Basal pneumonia due to unspecified organism | |
| 5324 | | H28..00 | | Atypical pneumonia | |
| 104121 | | H2B..00 | | Community acquired pneumonia | |
| 389 | | K15..00 | | Cystitis | |
| 15074 | | K150.00 | | Acute cystitis | |
| 1353 | | K155.00 | | Recurrent cystitis | |
| 22682 | | K15y000 | | Cystitis cystica | |
| 34645 | | K15y200 | | Abscess of bladder | |
| 1289 | | K190.00 | | Urinary tract infection, site not specified | |
| 1572 | | K190.11 | | Recurrent urinary tract infection | |
| 4453 | | K190100 | | Pyuria, site not specified | |
| 10515 | | K190300 | | Recurrent urinary tract infection | |
| 2985 | | K190311 | | Recurrent UTI | |
| 97002 | | K190500 | | Urinary tract infection | |
| 104141 | | K190600 | | Urosepsis | |
| 150 | | K190z00 | | Urinary tract infection, site not specified NOS | |
| 2465 | | K193.00 | | Urethral caruncle | |
| 507 | | K197.00 | | Haematuria | |
| 19361 | | K197.11 | | Traumatic haematuria | |
| 2784 | | K197200 | | Microscopic haematuria | |
| 7232 | | K197300 | | Frank haematuria | |
| 5264 | | K19y300 | | Pneumaturia | |
| 7733 | | K19y411 | | Urethral bleeding | |
| 885 | | A38..00 | | Septicaemia | |
| 30102 | | A381000 | | Septicaemia due to Staphylococcus aureus | |
| 10872 | | A384200 | | Escherichia coli septicaemia | |
| 23991 | | A384211 | | E.coli septicaemia | |
| 33765 | | A38z.00 | | Septicaemia NOS | |
| 2136 | | A38z.11 | | Sepsis | |
| 3382 | | A3B0.00 | | Streptococcal infection | |
| 1426 | | A3B1.00 | | Staphylococcal infection | |
| 8673 | | A3B1100 | | Meticillin resistant staphylococcus aureus | |
| 5534 | | A3B2.00 | | Pneumococcal infection | |
| 12062 | | A3B4.00 | | Escherichia coli infection | |
| 8329 | | A3B4.11 | | E.coli infection | |
| 6856 | | A3B7.00 | | Pseudomonas infection | |
| 105405 | | A3BC.00 | | Infection due to ESBL producing bacteria | |
| 5945 | | A3By800 | | Coliform bacteria | |
| 104028 | | A3C..00 | | Sepsis | |
| 104150 | | A3Cy.00 | | Other specified sepsis | |
| 4328 | | F4G0100 | | Orbital cellulitis | |
| 8852 | | F501112 | | Cellulitis, external ear | |
| 4456 | | K284300 | | Cellulitis of scrotum | |
| 4779 | | M020.00 | | Cellulitis and abscess of finger | |
| 3527 | | M020000 | | Cellulitis and abscess of finger unspecified | |
| 3960 | | M021.00 | | Cellulitis and abscess of toe | |
| 3363 | | M021000 | | Cellulitis and abscess of toe unspecified | |
| 16536 | | M03..00 | | Other cellulitis and abscess | |
| 3998 | | M030.00 | | Cellulitis and abscess of face | |
| 10485 | | M030111 | | Cellulitis and abscess of nose | |
| 1874 | | M032200 | | Cellulitis and abscess of back | |
| 14937 | | M032400 | | Cellulitis and abscess of umbilicus | |
| 3461 | | M033.00 | | Cellulitis and abscess of arm | |
| 1415 | | M034.11 | | Cellulitis and abscess of hand | |
| 2914 | | M034000 | | Cellulitis and abscess of hand unspecified | |
| 7865 | | M036.00 | | Cellulitis and abscess of leg excluding foot | |
| 10326 | | M036.11 | | Cellulitis and abscess of leg | |
| 25890 | | M036300 | | Cellulitis and abscess of lower leg | |
| 680 | | M036z00 | | Cellulitis and abscess of leg NOS | |
| 2089 | | M037000 | | Cellulitis and abscess of foot unspecified | |
| 7328 | | M037200 | | Cellulitis in diabetic foot | |
| 309 | | M03z.00 | | Cellulitis and abscess NOS | |
| 4207 | | M03z000 | | Cellulitis NOS | |
| 943 | | M05..00 | | Impetigo | |
| 14934 | | M05z.00 | | Impetigo NOS | |
| 6833 | | M08..00 | | Cutaneous cellulitis | |
| 1315 | | M081.00 | | [X]Cellulitis of other parts of limb | |
| 6368 | | M085.00 | | Cellulitis of leg | |
| 7684 | | M08B.00 | | Cellulitis of foot | |
| 94868 | | M08C.00 | | Cellulitis of toe | |

Read codes used to identify Ischaemic stroke / TIA

| **medcode** | **Read code** | **Read term** |
| --- | --- | --- |
| 8837 | G64..00 | Cerebral arterial occlusion |
| 5363 | G64..11 | CVA - cerebral artery occlusion |
| 569 | G64..12 | Infarction - cerebral |
| 6155 | G64..13 | Stroke due to cerebral arterial occlusion |
| 16517 | G640.00 | Cerebral thrombosis |
| 36717 | G640000 | Cerebral infarction due to thrombosis of cerebral arteries |
| 3149 | G64z.00 | Cerebral infarction NOS |
| 5602 | G64z.12 | Cerebellar infarction |
| 10504 | G64z300 | Right sided cerebral infarction |
| 504 | G65..00 | Transient cerebral ischaemia |
| 3132 | G65..11 | Drop attack |
| 1433 | G65..12 | Transient ischaemic attack |
| 1469 | G66..00 | Stroke and cerebrovascular accident unspecified |
| 1298 | G66..11 | CVA unspecified |
| 6253 | G66..12 | Stroke unspecified |
| 6116 | G66..13 | CVA - Cerebrovascular accident unspecified |
| 51767 | G666.00 | Pure sensory lacunar syndrome |
| 12833 | G668.00 | Right sided CVA |

Read codes used to identify venous thromboembolism

| **medcode** | **Read code** | **Read term** |
| --- | --- | --- |
| 94552 | 8HTm.00 | Referral to deep vein thrombosis clinic |
| 1266 | G401.00 | Pulmonary embolism |
| 9701 | G401.12 | Pulmonary embolus |
| 3576 | G801.00 | Deep vein phlebitis and thrombophlebitis of the leg |
| 824 | G801.11 | Deep vein thrombosis |
| 3392 | G801.13 | DVT - Deep vein thrombosis |
| 15382 | G801600 | Thrombophlebitis of the femoral vein |
| 22038 | G801D00 | Deep vein thrombosis of lower limb |
| 100103 | G824.00 | Axillary vein thrombosis |

**ICD-10 codes used to define the outcomes**

The following ICD-10 codes were used to identify the following outcomes in the HES or ONS data:

- Fracture – S02, S12, S22, S32, S42, S52, S62, S72, S82, or S92
- Hip fracture – S72.0, S27.1, or S72.2
- Forearm fracture – S52 or S62
- Fall – W0 or W1
- Acute bacterial infection – A4, B95, B96, H05.0, H60.1, K12.2, L03, J15, J18, N30.0, N30.2, N30.8, N30.9, or N39.0
- Ischaemic stroke/TIA – G45 or I63
- Venous thromboembolism - I26, I80, I81, or I82
