# Supplementary material for: Adverse effects of Z-drugs for sleep disturbance in people living with dementia: a population-based cohort study
Source: BMC Med. 2020 Nov 24;18:351. doi: 10.1186/s12916-020-01821-5 (PMC7683259; doi:10.1186/s12916-020-01821-5)
Supplement: Supplementary file 4 — Additional file 4. Study protocol registration, Definition of covariates and Multiple Imputation Methods. [file 12916_2020_1821_MOESM4_ESM.docx]

**Appendix file 4 – study protocol registration, Definition of covariates and Multiple Imputation Methods**

**Study protocol registration**

The study protocol was approved by the Independent Scientific Advisory Committee (ISAC) for CPRD research prior to data being released to the research team and was made available to journal reviewers (protocol number 16_181R). The study was registered on the ENCePP e-register of pharmacoepidemiology studies (register number EUPAS18006). Changes to the protocol include the exclusion of (1) the comparison to patients prescribed low-dose TCAs, as many patients were not prescribed them for sleep disturbance, (2) the urinary tract infection or respiratory infection outcome due to large overlap with the included acute infection outcome, and (3) the agitation/psychosis outcome as this was poorly recorded. To reduce complexity, the analysis of cumulative DDDs was omitted, but is available from the authors. We excluded midazolam injection from the benzodiazepine group due to its almost exclusive use in palliative care and strong association with mortality. A post-hoc decision was to censor at new antipsychotic prescription to reduce the likelihood that observed associations were due to new antipsychotic use. We examined average daily Z-drug dose over follow-up, but in post-hoc analysis, to reflect changes in dose, we examined time-varying daily DDDs.

**Definition of covariates**

We coded potentially confounding variables from CPRD (unless otherwise stated) on the index date.

- *Demographic factors:* Age, sex, year, residence (care home, lives alone at home, lives not alone at home) practice-level Index of Multiple Deprivation quintile, Strategic Health Authority region of England, index date, ethnicity (white, other)
- *Health behaviours:* Smoking (current smoker, ex-smoker, non-smoker), alcohol use (yes/no), Body Mass Index (kg/m2, most recent value in last 5 years), last systolic blood pressure (mmHg, most recent value in last 5 years).
- *Immunisations in last 12 months:* influenza, pneumonia
- *Dementia subtype and proxies for dementia severity:* dementia subtype (Alzheimer’s dementia, vascular dementia, mixed/other dementia, unspecified dementia), time since dementia diagnosis, cognitive enhancers in last 90 days, antipsychotic use in last 365 days, history of agitation/psychosis in dementia, end of life care (record of palliative care or end of life plans having been discussed).
- *Proxies for sleep disturbance severity:* sleep disturbance diagnosis before dementia diagnosis, previous Z-drug prescription (prior to 12 months before index date), previous benzodiazepine prescription (prior to 12 months before index date).
- *Comorbidities:* osteoporosis, other musculoskeletal conditions, depression, depression symptoms, anxiety, anxiety symptoms, Parkinson’s disease, urinary incontinence, age related macular degeneration, glaucoma, cataract, other visual impairment, diabetes, hyperlipidaemia, hypertension, heart attack, heart failure, atrial fibrillation, ischaemic stroke/TIA, angina, venous thromboembolism, osteoarthritis, rheumatoid arthritis, migraine/headache, back/neck pain, cancer, and Chronic Obstructive Pulmonary Disease.
- *Medical history in last 12 months:* number of GP consultations, hospital admissions (0, 1, or ≥2 using HES data), a fall, a fracture, LRTI/ UTI, dizziness/unsteadiness, and faints/syncope.
- *Concurrent medication use:* any prescription in the last 90 days of: Selective Serotonin Reuptake Inhibitors (SSRIs), non-SSRI or TCA antidepressants, other sedatives/hypnotics, antipsychotics, antihistamines, analgesics, antiepileptic drugs, anticoagulants, antiplatelets, cardiac glycosides, diuretics, calcium channel blockers, ACE inhibitors, angiotensin II receptor antagonists, beta blockers, lipid-regulating drugs, nonsteroidal anti-inflammatory drugs, bisphosphonates, diabetes drugs, inhaled corticosteroids, calcium/vitamin D, and any prescription in the last 30 days for antibiotics.

HES data was additionally used to supplement the coding of a history of falls, fractures, LRTI/UTI, ischaemic stroke/TIA, agitation/psychosis, venous thromboembolism, dementia subtype, ethnicity, and care home residence.

**Multiple Imputation Methods**

We assumed the missing at random assumption held and is reasonable. Multiple imputation by chained equations was performed using Stata’s‘mi impute’command to create 10 imputed datasets [1]. Body mass index splines and systolic blood pressure splines were imputed using linear regression [2], and ethnicity, care home residence, living alone, alcohol use, ever smoker, and current smoker using logistic regression. Imputation was performed separately for men and women. Each imputation model included all other variables being imputed as well as age, sex, all covariates listed above, the drug cohort, cumulative DDDs of Z-drug or benzodiazepine exposure, and the nelson-Aalen hazard and the event status for each outcome analysed [3]. Interaction terms were also included for drug cohort and death [4].

**References**

1 Stata Corporation. Stata statistical software: release 13. College Station, TX: StataCorp LP 2013.

2 Hippel PTV. How to Impute Interactions, Squares, and Other Transformed Variables. *Sociological Methodology* 2009;**39**:265–91. doi:10.1111/j.1467-9531.2009.01215.x

3 White IR, Royston P. Imputing missing covariate values for the Cox model. *Statistics in Medicine* 2009;**28**:1982–98. doi:10.1002/sim.3618

4 Tilling K, Williamson EJ, Spratt M, *et al.* Appropriate inclusion of interactions was needed to avoid bias in multiple imputation. *J Clin Epidemiol* 2016;**80**:107–15. doi:10.1016/j.jclinepi.2016.07.004
