# Supplementary material for: Adverse effects of Z-drugs for sleep disturbance in people living with dementia: a population-based cohort study
Source: BMC Med. 2020 Nov 24;18:351. doi: 10.1186/s12916-020-01821-5 (PMC7683259; doi:10.1186/s12916-020-01821-5)
Supplement: Supplementary file 5 — Additional file 5. Additional exclusion criteria, frequency of missing data, dose changes during follow-up, additional analysis results tables and flowchart of patient selection. [file 12916_2020_1821_MOESM5_ESM.docx]

**Additional file 5. Additional exclusion criteria, frequency of missing data, dose changes during follow-up, additional analysis results tables and flowchart of patient selection**

Table S1. Additional exclusion criteria for each outcome.

| Outcome | Additional exclusion criteria |
| --- | --- |
| Falls and fractures | Patients with a recorded fall or fracture in the 32 days before index date [22] |
| Infection and new antibiotic prescription | An infection or prescription for a medication for infection in the 30 days before the index date |
| Ischaemic stroke/TIA | Ischaemic stroke/TIA in the 30 days before the index date (post-hoc decision) |
| Venous thromboembolism | Venous thromboembolism in the 30 days before the index date (post-hoc decision) |
| New antidepressant prescription | Prescription for antidepressants in the 12 months before the index date |
| New antipsychotic prescription | Prescription for antipsychotics in the 12 months before the index date |

Table S2. Frequency of missing data within each exposure cohort of patients with dementia

|  | **Z-drug (n=3,532)** | | **Sleep disturbance, no sedative (n=1,833)** | | **No Z-drug, proximal GP consultation (n=10,214)** | | **Benzodiazepine (n=5,172)** | | **No benzodiazepine, proximal GP consultation (n=15,174)** | |
| --- | --- | --- | --- | --- | --- | --- | --- | --- | --- | --- |
| **Characteristic** | **n** | **%** | **n** | **%** | **n** | **%** | **n** | **%** | **n** | **%** |
| Ethnicity | 388 | 11% | 249 | 14% | 1214 | 12% | 627 | 12% | 1880 | 12% |
| Residence | 855 | 24% | 334 | 18% | 2279 | 22% | 1232 | 24% | 3552 | 23% |
| Smoking status | 141 | 4% | 48 | 3% | 336 | 3% | 186 | 4% | 497 | 3% |
| Alcohol use | 358 | 10% | 96 | 5% | 902 | 9% | 527 | 10% | 1274 | 8% |
| Body mass index | 1119 | 32% | 474 | 26% | 3033 | 30% | 1663 | 32% | 4336 | 29% |
| Systolic blood pressure | 103 | 3% | 27 | 1% | 230 | 2% | 182 | 4% | 351 | 2% |

Table S3. Defined Daily Doses (DDDs) of Z-drugs and benzodiazepines at index date and maximum during follow-up

|  | Z-drugs (n=3,532) | | Benzodiazepines (n=5,172) | |
| --- | --- | --- | --- | --- |
| DDD | At index date, n(%) | Maximum during follow-up, n(%) | At index date, n(%) | Maximum during follow-up, n(%) |
| <0.5 | 29 (0.8%) | 0 (0.0%) | 2751 (53.2%) | 2465 (47.7%) |
| 0.5 | 2743 (77.7%) | 2406 (68.1%) | 1352 (26.1%) | 1256 (24.3%) |
| 0.6-0.9 | 176 (5.0%) | 190 (5.4%) | 568 (11.0%) | 755 (14.6%) |
| ≥1* | 584 (16.5%) | 936 (26.5%) | 502 (9.7%) | 697 (13.5%) |

*Only six patients at index date and ten patients during follow-up were prescribed more than 1 DDD of Z-drugs (and were either prescribed 1.5 or 2 DDDs).

Table S4. Adjusted hazard ratios for new benzodiazepine prescription and adverse events for people with dementia

| Outcome | **Benzodiazepine (n=5,172)** | **Benzodiazepine vs no benzodiazepine (proximal GP consultation)** |
| --- | --- | --- |
| Fracture |  |  |
| Incidence rate per 100PY (events) | 12.5 (223) | 8.4 (349) |
| Age, sex adjusted HR (95% CI) | NA | 1.88 (1.70 to 2.08) |
| Fully adjusted HR (95% CI)^*^ | NA | 1.51 (1.35 to 1.69) |
| Hip fracture |  |  |
| Incidence rate per 100PY (events) | 5.1 (94) | 3.9 (163) |
| HR for Z (95% CI)a | NA | 1.32 (1.03 to 1.71) |
| Fully adjusted^a^ | NA | 1.17 (0.87 to 1.57) |
| Forearm/wrist/hand fracture |  |  |
| Incidence rate per 100PY (events) | 2.5 (46) | 1.5 (62) |
| HR for Z (95% CI)a | NA | 1.71 (1.14 to 2.55) |
| Fully adjusted^a^ | NA | 1.51 (0.99 to 2.32) |
| Fall |  |  |
| Incidence rate per 100PY (events) | 35.8 (585) | 26.7 (1027) |
| HR for Z (95% CI)a | NA | 1.39 (1.25 to 1.54) |
| Fully adjusted^a^ | NA | 1.27 (1.13 to 1.43) |
| Mortality |  |  |
| Incidence rate per 100PY (events) | 39.0 (736) | 21.7 (927) |
| HR for Z (95% CI)a | NA | 1.88 (1.70 to 2.08) |
| Fully adjusted^a^ | NA | 1.51 (1.35 to 1.69) |
| Acute bacterial infection |  |  |
| Incidence rate per 100PY (events) | 57.8 (371) | 39.2 (1842) |
| HR for Z (95% CI)a | NA | 1.19 (1.06 to 1.34) |
| Fully adjusted^a^ | NA | 1.08 (0.95 to 1.22) |
| Ischemic stroke/TIA |  |  |
| Incidence rate per 100PY (events) | 6.0 (110) | 5.2 (219) |
| HR for Z (95% CI)a | NA | 1.16 (0.92 to 1.46) |
| Fully adjusted^a^ | NA | 1.01 (0.77 to 1.31) |
| Venous thromboembolism |  |  |
| Incidence rate per 100PY (events) | 2.5 (47) | 1.7 (71) |
| HR for Z (95% CI)a | NA | 1.51 (1.04 to 2.20) |
| Fully adjusted^a^ | NA | 1.31 (0.84 to 2.04) |

Abbreviations: HR, hazard ratio; CI, confidence interval; GP, general practitioner; PY, person-years.

*Adjusted for all covariates listed in table 1.

** Fully-adjusted HR remaining statistically significant after controlling the false discovery rate to <5% (based on 13 outcomes)

Table S5. Adjusted hazard ratios for benzodiazepine prescription and adverse events for people with dementia according to prescribed Daily Defined Doses (DDDs) of benzodiazepines

| Outcome |  | **BZD (n=5,172) vs no benzodiazepine, proximal GP consultation (n=15,174)** | | | |  |  |  |  |  |  |  |  |  |  |  |  |  |  |  |  |
| --- | --- | --- | --- | --- | --- | --- | --- | --- | --- | --- | --- | --- | --- | --- | --- | --- | --- | --- | --- | --- | --- |
| and DDDs | No. | Age, sex adjusted | | Fully adjusted^a^ | |  |  |  |  |  |  |  |  |  |  |  |  |  |  |  |  |
| prescribed | events | HR | 95% CI | HR | 95% CI |  |  |  |  |  |  |  |  |  |  |  |  |  |  |  |  |
| Fracture |  |  |  |  |  |  |  |  |  |  |  |  |  |  |  |  |  |  |  |  |  |
| 0 | 351 | 1.00 |  | 1.00 |  |  |  |  |  |  |  |  |  |  |  |  |  |  |  |  |  |
| <0.5 | 119 | 1.55 | 1.26 to 1.91 | 1.45 | 1.15 to 1.82 |  |  |  |  |  |  |  |  |  |  |  |  |  |  |  |  |
| 0.5 | 51 | 1.25 | 0.92 to 1.68 | 1.13 | 0.82 to 1.55 |  |  |  |  |  |  |  |  |  |  |  |  |  |  |  |  |
| >0.5 | 53 | 1.82 | 1.36 to 2.45 | 1.74 | 1.26 to 2.41 |  |  |  |  |  |  |  |  |  |  |  |  |  |  |  |  |
| Hip fracture | |  |  |  |  |  |  |  |  |  |  |  |  |  |  |  |  |  |  |  |  |
| 0 | 164 | 1.00 |  | 1.00 |  |  |  |  |  |  |  |  |  |  |  |  |  |  |  |  |  |
| <0.5 | 54 | 1.47 | 1.08 to 2.00 | 1.34 | 0.95 to 1.88 |  |  |  |  |  |  |  |  |  |  |  |  |  |  |  |  |
| 0.5 | 17 | 0.86 | 0.52 to 1.41 | 0.76 | 0.45 to 1.29 |  |  |  |  |  |  |  |  |  |  |  |  |  |  |  |  |
| >0.5 | 23 | 1.67 | 1.07 to 2.59 | 1.45 | 0.89 to 2.36 |  |  |  |  |  |  |  |  |  |  |  |  |  |  |  |  |
| Forearm fracture | | | | | | | | | | | | | | | | | |  |  |  |  |
| 0 | 62 | 1.00 |  | 1.00 |  |  |  |  |  |  |  |  |  |  |  |  |  |  |  |  |  |
| <0.5 | 29 | 2.10 | 1.34 to 3.30 | 1.86 | 1.23 to 3.06 |  |  |  |  |  |  |  |  |  |  |  |  |  |  |  |  |
| 0.5 | 10 | 1.36 | 0.68 to 2.73 | 1.23 | 0.59 to 2.56 |  |  |  |  |  |  |  |  |  |  |  |  |  |  |  |  |
| >0.5 | 7 | 1.27 | 0.57 to 2.83 | 1.08 | 0.48 to 2.45 |  |  |  |  |  |  |  |  |  |  |  |  |  |  |  |  |
| Fall |  |  |  |  |  |  |  |  |  |  |  |  |  |  |  |  |  |  |  |  |  |
| 0 | 1040 | 1.00 |  | 1.00 |  |  |  |  |  |  |  |  |  |  |  |  |  |  |  |  |  |
| <0.5 | 311 | 1.43 | 1.26 to 1.62 | 1.29 | 1.12 to 1.49 |  |  |  |  |  |  |  |  |  |  |  |  |  |  |  |  |
| 0.5 | 146 | 1.23 | 1.03 to 1.47 | 1.13 | 0.94 to 1.37 |  |  |  |  |  |  |  |  |  |  |  |  |  |  |  |  |
| >0.5 | 126 | 1.53 | 1.26 to 1.85 | 1.44 | 1.18 to 1.77 |  |  |  |  |  |  |  |  |  |  |  |  |  |  |  |  |
| Mortality |  |  |  |  |  |  |  |  |  |  |  |  |  |  |  |  |  |  |  |  |  |
| 0 | 945 | 1.00 |  | 1.00 |  |  |  |  |  |  |  |  |  |  |  |  |  |  |  |  |  |
| <0.5 | 334 | 1.63 | 1.43 to 1.85 | 1.41 | 1.22 to 1.62 |  |  |  |  |  |  |  |  |  |  |  |  |  |  |  |  |
| 0.5 | 171 | 1.53 | 1.30 to 1.81 | 1.13 | 0.95 to 1.35 |  |  |  |  |  |  |  |  |  |  |  |  |  |  |  |  |
| >0.5 | 228 | 3.09 | 2.65 to 3.61 | 2.36 | 1.98 to 2.80 |  |  |  |  |  |  |  |  |  |  |  |  |  |  |  |  |
| Acute bacterial infection | | | | | | | | | | | | | | | | | | |  |  |  |
| 0 | 1007 | 1.00 |  | 1.00 |  |  |  |  |  |  |  |  |  |  |  |  |  |  |  |  |  |
| <0.5 | 274 | 1.30 | 1.14 to 1.49 | 1.14 | 0.98 to 1.32 |  |  |  |  |  |  |  |  |  |  |  |  |  |  |  |  |
| 0.5 | 153 | 1.40 | 1.18 to 1.67 | 1.22 | 1.01 to 1.47 |  |  |  |  |  |  |  |  |  |  |  |  |  |  |  |  |
| >0.5 | 125 | 1.68 | 1.39 to 2.03 | 1.52 | 1.24 to 1.86 |  |  |  |  |  |  |  |  |  |  |  |  |  |  |  |  |
| Ischemic stroke/transient ischemic attack | | | | | | | | | | | | | | | | | | | | |  |
| 0 | 221 | 1.00 |  | 1.00 |  |  |  |  |  |  |  |  |  |  |  |  |  |  |  |  |  |
| <0.5 | 45 | 0.93 | 0.67 to 1.28 | 0.88 | 0.61 to 1.27 |  |  |  |  |  |  |  |  |  |  |  |  |  |  |  |  |
| 0.5 | 34 | 1.26 | 0.88 to 1.81 | 0.94 | 0.64 to 1.39 |  |  |  |  |  |  |  |  |  |  |  |  |  |  |  |  |
| >0.5 | 30 | 1.59 | 1.09 to 2.33 | 1.37 | 0.91 to 2.08 |  |  |  |  |  |  |  |  |  |  |  |  |  |  |  |  |
| Venous thromboembolism | | | | | | | | | | | | | | | | | | |  |  |  |
| 0 | 71 | 1.00 |  | 1.00 |  |  |  |  |  |  |  |  |  |  |  |  |  |  |  |  |  |
| <0.5 | 26 | 1.65 | 1.05 to 2.60 | 1.33 | 0.78 to 2.28 |  |  |  |  |  |  |  |  |  |  |  |  |  |  |  |  |
| 0.5 | 12 | 1.35 | 0.73 to 2.49 | 1.25 | 0.65 to 2.41 |  |  |  |  |  |  |  |  |  |  |  |  |  |  |  |  |
| >0.5 | 9 | 1.41 | 0.70 to 2.86 | 1.16 | 0.54 to 2.52 |  |  |  |  |  |  |  |  |  |  |  |  |  |  |  |  |

Abbreviations: BZD, benzodiazepine; DDD, daily defined dose; HR, hazard ratio; CI, confidence interval; GP, general practitioner; PY, person-years.

*Adjusted for all covariates listed in table 1.

Table S6. Adjusted hazard ratios for benzodiazepine prescription and new prescriptions, GP consultations and hospital admissions for people with dementia according to prescribed Daily Defined Doses (DDDs) of benzodiazepines

|  |  | **Benzodiazepine (n=5,172) vs no benzodiazepine, proximal GP consultation (n=15,174)** | | | |  |  |  |  |  |  |  |  |  |  |  |  |  |  |  |  |  |  |  |  |
| --- | --- | --- | --- | --- | --- | --- | --- | --- | --- | --- | --- | --- | --- | --- | --- | --- | --- | --- | --- | --- | --- | --- | --- | --- | --- |
| Outcome and | No. of | Age, sex adjusted | | Fully adjusted^a^ | |  |  |  |  |  |  |  |  |  |  |  |  |  |  |  |  |  |  |  |  |
| daily defined dose prescribed | events | HR | 95% CI | HR | 95% CI |  |  |  |  |  |  |  |  |  |  |  |  |  |  |  |  |  |  |  |  |
| Incident antipsychotic prescription | | | | | | | | | | | | | | | | | | | | | | |  |  |  |
| 0 | 507 | 1.00 |  | 1.00 |  |  |  |  |  |  |  |  |  |  |  |  |  |  |  |  |  |  |  |  |  |
| 0.5 | 266 | 3.33 | 2.87 to 3.87 | 3.32 | 2.81 to 3.93 |  |  |  |  |  |  |  |  |  |  |  |  |  |  |  |  |  |  |  |  |
| 0.6-0.9 | 123 | 2.83 | 2.32 to 3.45 | 2.52 | 2.03 to 3.12 |  |  |  |  |  |  |  |  |  |  |  |  |  |  |  |  |  |  |  |  |
| 1+ | 142 | 4.95 | 4.10 to 5.99 | 4.34 | 3.62 to 5.44 |  |  |  |  |  |  |  |  |  |  |  |  |  |  |  |  |  |  |  |  |
| Incident antidepressant prescription | | | | | | | | | | | | | | | | | | | | | | |  |  |  |
| 0 | 276 | 1.00 |  | 1.00 |  |  |  |  |  |  |  |  |  |  |  |  |  |  |  |  |  |  |  |  |  |
| 0.5 | 142 | 3.65 | 2.98 to 4.46 | 3.57 | 2.83 to 4.50 |  |  |  |  |  |  |  |  |  |  |  |  |  |  |  |  |  |  |  |  |
| 0.6-0.9 | 57 | 2.35 | 1.78 to 3.12 | 2.52 | 1.86 to 3.43 |  |  |  |  |  |  |  |  |  |  |  |  |  |  |  |  |  |  |  |  |
| 1+ | 42 | 2.54 | 1.83 to 3.53 | 2.62 | 1.81 to 3.79 |  |  |  |  |  |  |  |  |  |  |  |  |  |  |  |  |  |  |  |  |
| Incident antibiotic prescription | | | | | | | | | | | | | | | | | | |  |  |  |  |  |  |  |
| 0 | 2240 | 1.00 |  | 1.00 |  |  |  |  |  |  |  |  |  |  |  |  |  |  |  |  |  |  |  |  |  |
| 0.5 | 588 | 1.38 | 1.26 to 1.51 | 1.20 | 1.08 to 1.32 |  |  |  |  |  |  |  |  |  |  |  |  |  |  |  |  |  |  |  |  |
| 0.6-0.9 | 307 | 1.48 | 1.32 to 1.67 | 1.33 | 1.17 to 1.52 |  |  |  |  |  |  |  |  |  |  |  |  |  |  |  |  |  |  |  |  |
| 1+ | 199 | 1.27 | 1.09 to 1.47 | 1.20 | 1.03 to 1.39 |  |  |  |  |  |  |  |  |  |  |  |  |  |  |  |  |  |  |  |  |
| Number of GP consultations | | | | | | | | | | | | | | | | | | | | | |  |  |  |  |
| 0 | 59094 | 1.00 |  | 1.00 |  |  |  |  |  |  |  |  |  |  |  |  |  |  |  |  |  |  |  |  |  |
| 0.5 | 14086 | 0.96 | 0.92 to 1.00 | 0.96 | 0.93 to 1.00 |  |  |  |  |  |  |  |  |  |  |  |  |  |  |  |  |  |  |  |  |
| 0.6-0.9 | 7697 | 0.96 | 0.91 to 1.02 | 1.01 | 0.96 to 1.06 |  |  |  |  |  |  |  |  |  |  |  |  |  |  |  |  |  |  |  |  |
| 1+ | 6225 | 1.12 | 1.05 to 1.19 | 1.13 | 1.08 to 1.19 |  |  |  |  |  |  |  |  |  |  |  |  |  |  |  |  |  |  |  |  |
| Number of hospital admissions | | | | | | | | | | | | | | | | | | | | | | |  |  |  |
| 0 | 4614 | 1.00 |  | 1.00 |  |  |  |  |  |  |  |  |  |  |  |  |  |  |  |  |  |  |  |  |  |
| 0.5 | 1205 | 1.09 | 1.00 to 1.19 | 1.02 | 0.93 to 1.12 |  |  |  |  |  |  |  |  |  |  |  |  |  |  |  |  |  |  |  |  |
| 0.6-0.9 | 677 | 1.18 | 1.05 to 1.33 | 1.13 | 1.00 to 1.27 |  |  |  |  |  |  |  |  |  |  |  |  |  |  |  |  |  |  |  |  |
| 1+ | 559 | 1.34 | 1.15 to 1.56 | 1.32 | 1.15 to 1.51 |  |  |  |  |  |  |  |  |  |  |  |  |  |  |  |  |  |  |  |  |

Abbreviations: HR, hazard ratio; CI, confidence interval; GP, general practitioner.

*Adjusted for all covariates listed in table 1.

Table S7. Adjusted hazard ratios for new Z-drug prescription and adverse events for people with dementia according to average daily defined dose (DDD) during follow-up

|  | N (events) | **No sedative, sleep disturbance (n=1,833)** | | | | **No Z-drug, proximal GP consultation (n=10,214)** | | | |  |  |  |  |  |
| --- | --- | --- | --- | --- | --- | --- | --- | --- | --- | --- | --- | --- | --- | --- |
| Outcome and | in Z-drug | Age, sex adjusted | | Fully adjusted* | | Age, sex adjusted | | Fully adjusted^*^ | |  |  |  |  |  |
| average DDDs | cohort | HR | 95% CI | HR | 95% CI | HR | 95% CI | HR | 95% CI |  |  |  |  |  |
| Fracture |  |  | |  |  |  | |  |  |  |  |  |  |  |
| 0 | NA | 1.00 |  | 1.00 |  | 1.00 |  | 1.00 |  |  |  |  |  |  |
| 0.1-0.4 | 607 (30) | 1.18 | 0.79 to 1.78 | 1.21 | 0.76 to 1.93 | 1.18 | 0.80 to 1.73 | 1.22 | 0.81 to 1.84 |  |  |  |  |  |
| 0.5 | 1173 (13) | 1.56 | 0.83 to 2.94 | 1.51 | 0.79 to 2.87 | 1.83 | 1.03 to 3.25 | 1.85 | 1.04 to 3.31 |  |  |  |  |  |
| 0.6-0.9 | 1119 (68) | 1.46 | 1.08 to 2.94 | 1.30 | 0.91 to 1.86 | 1.42 | 1.07 to 1.89 | 1.35 | 0.99 to 1.84 |  |  |  |  |  |
| 1+ | 534 (22) | 1.77 | 1.12 to 2.78 | 1.71 | 1.03 to 2.86 | 1.76 | 1.13 to 2.74 | 1.71 | 1.07 to 2.75 |  |  |  |  |  |
| Hip fracture |  |  | |  |  |  |  |  |  |  |  |  |  |  |
| 0 | NA | 1.00 |  | 1.00 |  | 1.00 |  | 1.00 |  |  |  |  |  |  |
| 0.1-0.4 | 607 (16) | 1.38 | 0.78 to 2.42 | 1.60 | 0.83 to 3.08 | 1.39 | 0.81 to 2.38 | 1.51 | 0.82 to 2.80 |  |  |  |  |  |
| 0.5 | 1173 (5) | 1.33 | 0.49 to 3.59 | 1.21 | 0.44 to 3.36 | 1.78 | 0.71 to 4.44 | 1.83 | 0.72 to 4.67 |  |  |  |  |  |
| 0.6-0.9 | 1119 (33) | 1.53 | 0.99 to 2.36 | 1.22 | 0.74 to 2.02 | 1.52 | 1.01 to 2.29 | 1.31 | 0.81 to 2.12 |  |  |  |  |  |
| 1+ | 534 (14) | 2.38 | 1.33 to 4.26 | 1.95 | 0.99 to 3.85 | 2.47 | 1.40 to 4.35 | 2.17 | 1.12 to 4.21 |  |  |  |  |  |
| Forearm fracture | | | | | | |  |  |  |  |  |  |  |  |
| 0 | NA | 1.00 |  | 1.00 |  | 1.00 |  | 1.00 |  |  |  |  |  |  |
| 0.1-0.5 | 1780 (8) | 1.78 | 0.71 to 4.46 | 1.41 | 0.42 to 4.71 | 1.47 | 0.69 to 3.14 | 1.57 | 0.70 to 3.55 |  |  |  |  |  |
| 0.6+ | 1637 (16) | 1.92 | 0.97 to 3.81 | 1.84 | 0.69 to 4.93 | 1.46 | 0.79 to 2.69 | 1.80 | 0.84 to 3.87 |  |  |  |  |  |
| Fall |  |  |  |  |  |  |  |  |  |  |  |  |  |  |
| 0 | NA | 1.00 |  | 1.00 |  | 1.00 |  | 1.00 |  |  |  |  |  |  |
| 0.1-0.4 | 586 (97) | 1.14 | 0.91 to 1.43 | 1.07 | 0.83 to 1.37 | 1.61 | 1.29 to 2.01 | 1.60 | 1.26 to 2.02 |  |  |  |  |  |
| 0.5 | 1125 (45) | 1.26 | 0.91 to 1.76 | 1.22 | 0.86 to 1.72 | 1.94 | 1.43 to 2.65 | 1.94 | 1.41 to 2.68 |  |  |  |  |  |
| 0.6-0.9 | 1082 (182) | 1.14 | 0.95 to 1.37 | 1.02 | 0.83 to 1.25 | 1.61 | 1.35 to 1.91 | 1.48 | 1.22 to 1.79 |  |  |  |  |  |
| 1+ | 507 (61) | 1.30 | 0.99 to 1.71 | 1.21 | 0.89 to 1.64 | 1.87 | 1.42 to 2.46 | 1.77 | 1.31 to 2.38 |  |  |  |  |  |
| Mortality |  |  |  |  |  |  |  |  |  |  |  |  |  |  |
| 0 | NA | 1.00 |  | 1.00 |  | 1.00 |  | 1.00 |  |  |  |  |  |  |
| 0.1-0.4 | 619 (36) | 0.61 | 0.44 to 0.86 | 0.55 | 0.37 to 0.80 | 0.47 | 0.34 to 0.64 | 0.41 | 0.29 to 0.59 |  |  |  |  |  |
| 0.5 | 1206 (49) | 3.20 | 2.22 to 4.61 | 3.35 | 2.27 to 4.96 | 1.99 | 1.46 to 2.70 | 2.09 | 1.52 to 2.88 |  |  |  |  |  |
| 0.6-0.9 | 1157 (119) | 1.06 | 0.86 to 1.32 | 0.90 | 0.70 to 1.17 | 0.81 | 0.67 to 0.98 | 0.69 | 0.55 to 0.86 |  |  |  |  |  |
| 1+ | 550 (47) | 1.48 | 1.08 to 2.02 | 1.12 | 0.79 to 1.60 | 1.13 | 0.84 to 1.52 | 0.89 | 0.64 to 1.22 |  |  |  |  |  |
| Acute bacterial infection | | | | | | |  |  |  |  |  |  |  |  |
| 0 | NA | 1.00 |  | 1.00 |  | 1.00 |  | 1.00 |  |  |  |  |  |  |
| 0.1-0.4 | 203 (50) | 1.15 | 0.85 to 1.56 | 1.00 | 0.70 to 1.41 | 1.27 | 0.95 to 1.68 | 1.16 | 0.84 to 1.60 |  |  |  |  |  |
| 0.5 | 416 (28) | 1.70 | 1.09 to 2.66 | 1.52 | 0.93 to 2.47 | 1.91 | 1.29 to 2.83 | 1.91 | 1.24 to 2.93 |  |  |  |  |  |
| 0.6-0.9 | 434 (96) | 0.97 | 0.76 to 1.24 | 0.92 | 0.69 to 1.22 | 1.07 | 0.86 to 1.34 | 1.01 | 0.78 to 1.29 |  |  |  |  |  |
| 1+ | 188 (37) | 1.43 | 0.98 to 2.09 | 1.36 | 0.88 to 2.09 | 1.53 | 1.06 to 2.23 | 1.40 | 0.94 to 2.09 |  |  |  |  |  |
| Ischemic stroke/transient ischemic attack | | | | | | | | |  |  |  |  |  |  |
| 0 | NA | 1.00 |  | 1.00 |  | 1.00 |  | 1.00 |  |  |  |  |  |  |
| 0.1-0.4 | 609 (14) | 0.96 | 0.53 to 1.72 | 0.92 | 0.45 to 1.88 | 0.88 | 0.51 to 1.52 | 0.91 | 0.45 to 1.88 |  |  |  |  |  |
| 0.5 | 1191 (7) | 1.81 | 0.77 to 4.22 | 2.08 | 0.86 to 5.02 | 1.48 | 0.68 to 3.24 | 1.59 | 0.86 to 5.02 |  |  |  |  |  |
| 0.6-0.9 | 1142 (38) | 1.35 | 0.91 to 2.00 | 1.28 | 0.75 to 2.17 | 1.23 | 0.86 to 1.76 | 1.13 | 0.75 to 2.17 |  |  |  |  |  |
| 1+ | 542 (15) | 1.92 | 1.09 to 3.41 | 1.62 | 0.81 to 3.26 | 1.72 | 1.00 to 2.95 | 1.42 | 0.81 to 3.26 |  |  |  |  |  |
| Venous thromboembolism | | | | | | |  |  |  |  |  |  |  |  |
| 0 | NA | 1.00 |  | 1.00 |  | 1.00 |  | 1.00 |  |  |  |  |  |  |
| 0.1-0.5 | 1812 (11) | 2.23 | 1.12 to 4.45 | 2.34 | 0.95 to 5.77 | 1.73 | 0.90 to 3.31 | 1.48 | 0.64 to 3.40 |  |  |  |  |  |
| 0.6+ | 1703 (12) | 1.17 | 0.59 to 2.32 | 1.20 | 0.39 to 3.73 | 1.00 | 0.52 to 1.89 | 1.21 | 0.57 to 2.57 |  |  |  |  |  |

Abbreviations: HR, hazard ratio; CI, confidence interval; GP, general practitioner; PY, person-years.

*Adjusted for all covariates listed in table 1.

Table S8. Adjusted hazard ratios for new Z-drug prescription and new prescriptions and healthcare utilisation for people with dementia according to average daily defined dose (DDD) during follow-up

|  | N (events) | **No sedative, sleep disturbance (n=1,833)** | | | | **No Z-drug, proximal GP consultation (n=10,214)** | | | |  |  |  |  |  |
| --- | --- | --- | --- | --- | --- | --- | --- | --- | --- | --- | --- | --- | --- | --- |
| Outcome and | in Z-drug | Age, sex adjusted | | Fully adjusted^a^ | | Age, sex adjusted | | Fully adjusted^a^ | |  |  |  |  |  |
| average DDDs | cohort | HR | 95% CI | HR | 95% CI | HR | 95% CI | HR | 95% CI |  |  |  |  |  |
| Incident antipsychotic prescription | | | | | | | |  |  |  |  |  |  |  |
| 0 | NA | 1.00 |  | 1.00 |  | 1.00 |  | 1.00 |  |  |  |  |  |  |
| 0.1-0.4 | 461 (41) | 1.42 | 1.00 to 2.03 | 1.38 | 0.92 to 2.05 | 2.34 | 1.64 to 3.32 | 2.41 | 1.65 to 3.50 |  |  |  |  |  |
| 0.5 | 958 (33) | 1.85 | 1.21 to 2.81 | 1.83 | 1.18 to 2.84 | 3.50 | 2.38 to 5.14 | 3.70 | 2.46 to 5.56 |  |  |  |  |  |
| 0.6-0.9 | 790 (117) | 2.42 | 1.88 to 3.13 | 2.28 | 1.68 to 3.10 | 3.93 | 3.06 to 5.04 | 4.05 | 3.05 to 5.37 |  |  |  |  |  |
| 1+ | 338 (50) | 3.76 | 2.70 to 5.25 | 3.32 | 2.27 to 4.85 | 6.31 | 4.57 to 8.72 | 6.04 | 4.17 to 8.73 |  |  |  |  |  |
| Incident antidepressant prescription | | | | | | | |  |  |  |  |  |  |  |
| 0 | NA | 1.00 |  | 1.00 |  | 1.00 |  | 1.00 |  |  |  |  |  |  |
| 0.1-0.4 | 319 (28) | 1.92 | 1.24 to 2.97 | 2.14 | 1.27 to 3.58 | 2.01 | 1.34 to 3.02 | 2.27 | 1.43 to 3.60 |  |  |  |  |  |
| 0.5 | 659 (22) | 3.44 | 2.01 to 5.88 | 3.18 | 1.72 to 5.86 | 4.33 | 2.71 to 6.92 | 4.33 | 2.63 to 7.10 |  |  |  |  |  |
| 0.6-0.9 | 630 (68) | 2.37 | 1.71 to 3.29 | 2.41 | 1.59 to 3.65 | 2.53 | 1.88 to 3.40 | 2.87 | 2.05 to 4.00 |  |  |  |  |  |
| 1+ | 274 (16) | 2.02 | 1.17 to 3.50 | 2.46 | 1.31 to 4.64 | 2.24 | 1.32 to 3.80 | 3.05 | 1.70 to 5.48 |  |  |  |  |  |
| Incident antibiotic prescription | | | | | | | |  |  |  |  |  |  |  |
| 0 | NA | 1.00 |  | 1.00 |  | 1.00 |  | 1.00 |  |  |  |  |  |  |
| 0.1-0.4 | 410 (155) | 1.49 | 1.25 to 1.79 | 1.32 | 1.07 to 1.61 | 1.32 | 1.12 to 1.55 | 1.21 | 1.02 to 1.44 |  |  |  |  |  |
| 0.5 | 816 (80) | 1.55 | 1.20 to 2.01 | 1.48 | 1.12 to 1.93 | 1.36 | 1.08 to 1.70 | 1.31 | 1.03 to 1.66 |  |  |  |  |  |
| 0.6-0.9 | 804 (285) | 1.44 | 1.24 to 1.67 | 1.31 | 1.11 to 1.55 | 1.28 | 1.12 to 1.45 | 1.17 | 1.02 to 1.34 |  |  |  |  |  |
| 1+ | 404 (99) | 1.57 | 1.25 to 1.97 | 1.41 | 1.10 to 1.79 | 1.38 | 1.12 to 1.71 | 1.27 | 1.01 to 1.59 |  |  |  |  |  |
| Number of GP consultations | | | | | | |  |  |  |  |  |  |  |  |
| 0 | NA | 1.00 |  | 1.00 |  | 1.00 |  | 1.00 |  |  |  |  |  |  |
| 0.1-0.4 | 619 (5141) | 1.24 | 1.14 to 1.35 | 1.14 | 1.06 to 1.22 | 0.92 | 0.86 to 0.99 | 1.04 | 0.98 to 1.11 |  |  |  |  |  |
| 0.5 | 1206 (3993) | 1.34 | 1.24 to 1.45 | 1.16 | 1.09 to 1.24 | 1.01 | 0.94 to 1.08 | 1.07 | 1.01 to 1.13 |  |  |  |  |  |
| 0.6-0.9 | 1157 (9202) | 1.27 | 1.18 to 1.36 | 1.16 | 1.09 to 1.24 | 0.95 | 0.89 to 1.01 | 1.06 | 1.01 to 1.12 |  |  |  |  |  |
| 1+ | 550 (2956) | 1.32 | 1.19 to 1.46 | 1.26 | 1.16 to 1.38 | 1.00 | 0.91 to 1.09 | 1.17 | 1.08 to 1.27 |  |  |  |  |  |
| Number of hospital admissions | | | | | | | |  |  |  |  |  |  |  |
| 0 | NA | 1.00 |  | 1.00 |  | 1.00 |  | 1.00 |  |  |  |  |  |  |
| 0.1-0.4 | 619 (432) | 1.21 | 1.04 to 1.42 | 1.18 | 1.01 to 1.38 | 1.13 | 0.98 to 1.29 | 1.10 | 0.95 to 1.26 |  |  |  |  |  |
| 0.5 | 1206 (456) | 1.62 | 1.40 to 1.87 | 1.61 | 1.39 to 1.85 | 1.51 | 1.33 to 1.71 | 1.46 | 1.29 to 1.66 |  |  |  |  |  |
| 0.6-0.9 | 1157 (783) | 1.21 | 1.01 to 1.44 | 1.05 | 0.91 to 1.21 | 1.15 | 0.95 to 1.39 | 0.99 | 0.87 to 1.12 |  |  |  |  |  |
| 1+ | 550 (273) | 1.37 | 1.13 to 1.66 | 1.28 | 1.06 to 1.54 | 1.31 | 1.09 to 1.56 | 1.18 | 1.00 to 1.40 |  |  |  |  |  |

Abbreviations: HR, hazard ratio; CI, confidence interval; GP, general practitioner; PY, person-years.

*Adjusted for all covariates listed in table 1.

Table S9. Adjusted hazard ratios for new Z-drug prescription and adverse events, new prescriptions and healthcare utilisation for people with dementia compared to sleep disturbance (with no mention of more than 6 hours sleep per night)

|  | **HR (95% CI) for Z-drug** | |
| --- | --- | --- |
| Outcome | **Age, sex adjusted** | **Fully adjusted*** |
| Fracture | 1.30 (1.01 to 1.68) | 1.21 (0.90 to 1.61) |
| Hip fracture | 1.41 (0.98 to 2.03) | 1.22 (0.81 to 1.85) |
| Forearm fracture | 1.70 (0.88 to 3.29) | 1.36 (0.56 to 3.26) |
| Fall | 1.06 (0.92 to 1.23) | 0.99 (0.84 to 1.17) |
| Mortality | 1.42 (1.21 to 1.67) | 1.30 (1.07 to 1.56) |
| Acute bacterial infection | 1.07 (0.88 to 1.29) | 0.97 (0.78 to 1.21) |
| Ischemic stroke/TIA | 1.31 (0.94 to 1.84) | 1.32 (0.85 to 2.03) |
| Venous thromboembolism | 1.53 (0.89 to 2.63) | 1.74 (0.77 to 3.92) |
| Incident antipsychotic prescription | 2.24 (1.80 to 2.80) | 2.08 (1.62 to 2.67) |
| Incident antidepressant prescription | 2.05 (1.54 to 2.73) | 2.08 (1.46 to 2.96) |
| Incident antibiotic prescription | 1.40 (1.24 to 1.58) | 1.31 (1.14 to 1.50) |
| Number of GP consultations** | 1.25 (1.18 to 1.32) | 1.16 (1.11 to 1.22) |
| Number of hospital admissions** | 1.29 (1.15 to 1.44) | 1.22 (1.09 to 1.36) |

Abbreviations: HR, hazard ratio; CI, confidence interval; GP, general practitioner.

*Adjusted for all covariates listed in table 1.

** Rate ratio (95% confidence interval)

Table S10. Adjusted hazard ratios for Z-drug prescription and adverse events, new prescriptions and healthcare utilisation for people with dementia compared to sleep disturbance (with no mention of more than 6 hours sleep per night), according to prescribed daily dose of Z-drugs

| Outcome and | Age, sex adjusted | | Fully adjusted^a^ | |
| --- | --- | --- | --- | --- |
| prescribed daily dose | HR | 95% CI | HR | 95% CI |
| Fracture |  |  |  |  |
| 0 | 1.00 |  | 1.00 |  |
| ≤0.5 | 1.22 | 0.92 to 1.60 | 1.12 | 0.82 to 1.53 |
| 0.7-0.8 | 1.19 | 0.47 to 2.98 | 1.01 | 0.37 to 2.75 |
| 1-2 | 1.59 | 1.11 to 2.28 | 1.54 | 1.04 to 2.28 |
| Hip fracture |  |  |  |  |
| 0 | 1.00 |  | 1.00 |  |
| ≤0.5 | 1.20 | 0.80 to 1.80 | 1.08 | 0.69 to 1.70 |
| 0.6-0.9 | 1.00 | 0.24 to 4.26 | 0.65 | 0.12 to 3.55 |
| 1+ | 2.13 | 1.33 to 3.40 | 1.75 | 1.03 to 2.98 |
| Forearm fracture |  |  |  |  |
| 0 | 1.00 |  | 1.00 |  |
| ≤0.5 | 1.56 | 0.75to 3.27 | 1.14 | 0.44 to 2.97 |
| 0.7+ | 2.07 | 0.92 to 4.68 | 1.91 | 0.67 to 5.43 |
| Fall |  |  |  |  |
| 0 | 1.00 |  | 1.00 |  |
| 0.5 | 0.99 | 0.85 to 1.16 | 0.92 | 0.77 to 1.10 |
| 0.6-0.9 | 0.80 | 0.47 to 1.36 | 0.69 | 0.39 to 1.22 |
| 1+ | 1.34 | 1.09 to 1.66 | 1.28 | 1.02 to 1.60 |
| Mortality |  |  |  |  |
| 0 | 1.00 |  | 1.00 |  |
| 0.5 | 1.41 | 1.18 to 1.67 | 1.30 | 1.07 to 1.59 |
| 0.6-0.9 | 1.45 | 0.88 to 2.39 | 1.51 | 0.90 to 2.54 |
| 1+ | 1.47 | 1.16 to 1.85 | 1.26 | 0.97 to 1.63 |
| Acute bacterial infection | |  |  |  |
| 0 | 1.00 |  | 1.00 |  |
| 0.5 | 1.10 | 0.93 to 1.31 | 0.96 | 0.80 to 1.16 |
| 0.6-0.9 | 0.94 | 0.53 to 1.65 | 0.84 | 0.46 to 1.54 |
| 1+ | 1.35 | 1.07 to 1.70 | 1.21 | 0.94 to 1.55 |
| Ischemic stroke/transient ischemic attack | | |  |  |
| 0 | 1 |  | 1.00 |  |
| 0.5 | 1.04 | 0.70 to 1.52 | 1.08 | 0.68 to 1.73 |
| 0.6-0.9 | 1.96 | 0.77 to 4.96 | 1.94 | 0.73 o 5.11 |
| 1+ | 2.07 | 1.35 to 3.18 | 1.83 | 1.09 to 3.08 |
| Venous thromboembolism | |  |  |  |
| 0 | 1.00 |  | 1.00 |  |
| ≤0.5 | 1.88 | 1.08 to 3.30 | 2.10 | 0.93 to 4.77 |
| 0.7+ | 0.72 | 0.25 to 2.10 | 0.93 | 0.24 to 3.53 |
| Incident antipsychotic prescription | | |  |  |
| 0 | 1.00 |  | 1.00 |  |
| 0.5 | 1.84 | 1.45 to 2.33 | 1.75 | 1.35 to 2.28 |
| 0.6-0.9 | 2.97 | 1.81 to 4.88 | 2.37 | 1.36 to 4.16 |
| 1+ | 3.60 | 2.75 to 4.72 | 3.14 | 2.31 to 4.28 |
| Incident antidepressant prescription | | |  |  |
| 0 | 1.00 |  | 1.00 |  |
| 0.5 | 1.95 | 1.44 to 2.64 | 1.92 | 1.32 to 2.81 |
| 0.6-0.9 | 2.27 | 1.10 to 4.71 | 1.87 | 0.77 to 4.57 |
| 1+ | 2.36 | 1.58 to 3.52 | 2.75 | 1.72 to 4.39 |
| Incident antibiotic prescription | |  |  |  |
| 0 | 1.00 |  | 1.00 |  |
| 0.5 | 1.37 | 1.21 to 1.56 | 1.28 | 1.11 to 1.47 |
| 0.6-0.9 | 1.31 | 0.89 to 1.92 | 1.18 | 0.78 to 1.80 |
| 1+ | 1.50 | 1.26 to 1.80 | 1.43 | 1.19 to 1.73 |
| Number of GP consultations** |  |  |  |  |
| 0 | 1.00 |  | 1.00 |  |
| 0.5 | 1.18 | 1.11 to 1.26 | 1.10 | 1.04 to 1.16 |
| 0.6-0.9 | 1.10 | 0.93 to 1.28 | 1.09 | 0.95 to 1.26 |
| 1+ | 1.19 | 1.09 to 1.30 | 1.16 | 1.07 to 1.25 |
| Number of hospital admissions** | | |  |  |
| 0 | 1.00 |  | 1.00 |  |
| 0.5 | 1.19 | 1.06 to 1.35 | 1.14 | 1.01 to 1.28 |
| 0.6-0.9 | 1.02 | 0.73 to 1.44 | 1.18 | 0.85 to 1.64 |
| 1+ | 1.30 | 1.10 to 1.52 | 1.24 | 1.06 to 1.46 |

Abbreviations: HR, hazard ratio; CI, confidence interval; GP, general practitioner.

*Adjusted for all covariates listed in table 1.

** Rate ratio (95% confidence interval)

Table S11. Adjusted hazard ratios for new Z-drug prescription and adverse events, new prescriptions and healthcare utilisation for people with dementia compared to new benzodiazepine prescription likely for sleep disturbance

|  | **HR (95% CI) for Z-drug** | |
| --- | --- | --- |
| Outcome | **Age, sex adjusted** | **Fully adjusted*** |
| Fracture | 0.94 (0.72 to 1.23) | 0.99 (0.73 to 1.34) |
| Hip fracture | 1.15 (0.77 to 1.71) | 1.19 (0.74 to 1.91) |
| Forearm fracture | 0.96 (0.51 to 1.83) | 1.23 (0.49 to 3.07) |
| Fall | 1.06 (0.90 to 1.25) | 1.03 (0.86 to 1.23) |
| Mortality | 0.81 (0.69 to 0.95) | 0.84 (0.70 to 1.00) |
| Acute bacterial infection | 0.96 (0.78 to 1.18) | 0.86 (0.68 to 1.09) |
| Ischemic stroke/TIA | 0.92 (0.65 to 1.31) | 1.17 (0.77 to 1.77) |
| Venous thromboembolism | 0.82 (0.45 to 1.48) | 0.57 (0.29 to 1.11) |
| Incident antipsychotic prescription | 0.79 (0.68 to 0.98) | 0.96 (0.78 to 1.17) |
| Incident antidepressant prescription | 1.26 (0.95 to 1.67) | 1.18 (0.85 to 1.65) |
| Incident antibiotic prescription | 0.93 (0.82 to 1.05) | 0.92 (0.81 to 1.05) |
| Number of GP consultations** | 0.98 (0.92 to 1.04) | 0.95 (0.90 to 1.00) |
| Number of hospital admissions** | 0.97 (0.85 to 1.11) | 0.90 (0.80 to 1.02) |

Abbreviations: HR, hazard ratio; CI, confidence interval; GP, general practitioner.

*Adjusted for all covariates listed in table 1.

** Rate ratio (95% confidence interval)

Figure S1. Selection of five cohort of patients with dementia from the Clinical Practice Research Datalink

Patients with dementia diagnosed when aged 55 years or older between January 2000 and March 2016 (n=64,103)

Patients in CPRD database July 2017 (n=17,148,866)

Patients with HES linkage (n=8,328,954)

Patients with ≥ 90 days of Up-To-Standard data history before dementia diagnosis (n=62,554)

Patients not diagnosed with severe mental illness or Down’s syndrome before dementia diagnosis (n=60,773)

Patients with follow-up after 365 days of data history (n=60,510)

Patients censored at first sleep apnoea, sleep related respiratory failure, or alcohol abuse record (n=59,505)

Patients with follow-up after 365 days without prescription of sedative-hypnotics (n=51,117)

Patients with no new prescription of other sedatives, low dose TCAs, or antipsychotics on index date (n=3,532)

Patients not prescribed sedative- hypnotics in last 365 days (n=4,038)

Patients first prescribed Z-drugs after dementia, by March 2016 (n=7,522)

Patients with no new prescription of other sedatives, low dose TCAs, or antipsychotics on index date (n=6,019)

Patients not prescribed sedative-hypnotics in last 365 days (n=7,220)

Patients prescribed benzodiazepines after dementia and by March 2016 (n=12,317)

Patients with ‘sleep disturbance’ between dementia diagnosis and March 2016 (n=1,833)

Patients matched with no Z-drug, at proximal GP consultation (n=10,214)

Patients matched with no benzodiazepine, at proximal GP consultation (n=15,174)

Patients not prescribed midazolam (n=5,172)
